# Diphenyl Diselenide and Temozolomide: Downregulation of Inflammatory, Redox, and Tumor-Associated Pathways in Glioblastoma

**DOI:** 10.1007/s12011-026-05064-y

**Published:** 2026-03-29

**Authors:** Guilherme Schmitt Rieder, Alessandra Schmitt Rieder, Lucas dos Santos da Silva, Babajide Oluwaseun Ajayi, José Cláudio Fonseca Moreira, Angela T. S. Wyse, Diogo Onofre Souza, Debora Guerini de Souza, João Batista Teixeira da Rocha

**Affiliations:** 1 Programa de Pós-Graduação em Ciências Biológicas: Bioquímica, ICBS, Universidade Federal do Rio Grande do Sul (UFRGS), Porto Alegre, Rio Grande do Sul Brasil; 2 Departamento de Bioquímica, ICBS, Universidade Federal do Rio Grande do Sul (UFRGS), Porto Alegre, Rio Grande do Sul Brasil; 3 Instituto do Cérebro do Rio Grande do Sul, Pontifícia Universidade Católica do Rio Grande do Sul (PUCRS), Porto Alegre, Rio Grande do Sul Brasil; 4 Departamento de Bioquímica e Biologia Molecular, Universidade Federal de Santa Maria (UFSM), Rio Grande do Sul, Santa Maria, Brasil

**Keywords:** Glioblastoma, Temozolomide, Diphenyl Diselenide, Antioxidant response, Inflammation

## Abstract

Glioblastoma (GBM) is the most common and lethal primary brain tumor, originating from glial cells and classified as grade IV by the World Health Organization (WHO). Standard treatment includes resection, radiotherapy, and the use of temozolomide (TMZ), a DNA-alkylating agent; however, therapeutic success is limited due to tumor heterogeneity and resistance mechanisms. Selenium (Se) is an essential element involved in selenoprotein formation, with reported anti-inflammatory, antioxidant, and antitumor activities. Among organoselenium compounds (OrganoSe), Diphenyl Diselenide (DPDS) has demonstrated antitumor, antioxidant, and anti-inflammatory properties, but its effects in human GBM are underexplored. Here, we investigated, for the first time, the antitumor capacity of DPDS, both alone and in combination with TMZ, in U87-MG cells. We evaluated cell viability, density, migration, biochemical and molecular pathways, including oxidative stress, inflammation, apoptosis, hypoxia response, epigenetic regulation, cell cycle, DNA damage response, and epithelial–mesenchymal transition (EMT). DPDS (20 µM) in combination with TMZ (100 µM) reduced cell viability and density. Co-exposure decreased cellular migration and oxidative stress markers (ROS production, SOD, and CAT activities). Gene expression analysis revealed downregulation of genes related to redox defense and inflammatory signaling, such as NRF2, KEAP1, GPX1, GPX4, TRXR1, SELENOS, SELENOK, SELENOI, NFKB1, IL6, IL-1B, TNFα, PTGS2, BAX, BAD, BCL-2, HIF1A, HDAC4, NEK1, NEK2, and SNAIL1. The results indicate that DPDS, particularly in combination with TMZ, attenuated pro-inflammatory and antioxidant profiles and interfered with pathways critical for GBM survival.

**Graphical Abstract:**

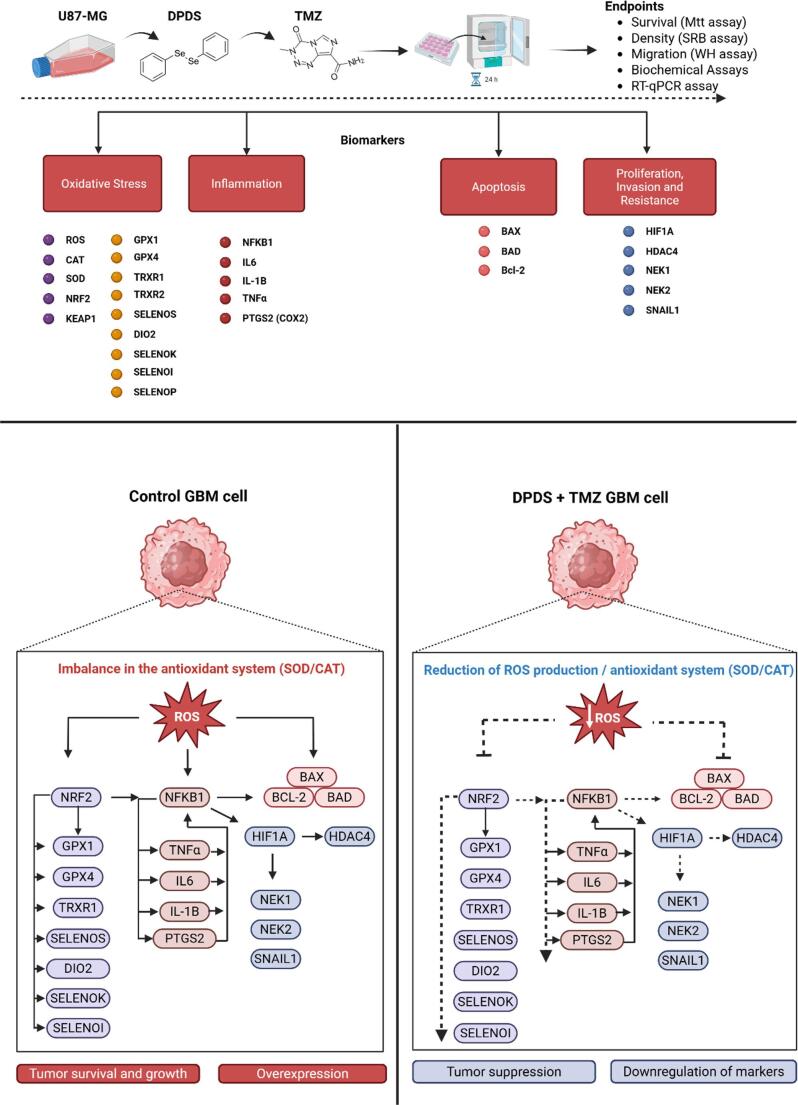

## Introduction

Brain tumors have high morbidity and mortality due to their location and invasive growth [1–3]. Glioblastoma is classified by the WHO as the highest grade of malignancy (grade IV) [3, 4]. It is one of the most lethal types of brain tumors and the most common neoplasm of the central nervous system (CNS) [3–6].

Temozolomide (TMZ) is one of the most commonly employed chemotherapeutic agents for treating glioblastoma. TMZ acts as a DNA alkylating agent, leading to cellular apoptosis [7, 8] and has proven effective when glioma is diagnosed early [9, 10]. However, several mechanisms of TMZ chemoresistance have already been observed [11, 12]. These resistance mechanisms underscore the urgent need to develop additional or complementary therapies to treat gliomas [6, 13]. Selenium (Se) is an essential element for human life as a component of selenoproteins [14–16]. These proteins modulate several biochemical processes, including anti-inflammatory, antioxidant, antiviral, and antitumor activities [17–19]. However, the exact role of selenoproteins in cancer development and progression remains poorly understood. For instance, glutathione peroxidases (GPXs) and thioredoxin reductases (TXNRDs) can be overexpressed in several tumor types, suggesting a dual role of these enzymes in both protecting cells from oxidative stress and potentially supporting tumor cell survival [20–25]. In fact, the study of selenoproteins expression in different types of cancer cells is still incipient.

Selenium-containing compounds have been investigated for their effects on glioblastoma for some time. Sodium selenite (inorganic selenium compound) and selenomethionine (a selenium-containing amino acid) were able to induce cell death in different cell lines of glioblastoma [26, 27]. NSAID–Ebselen hybrid derivatives have been reported as effective TrxR1 (TXNRD1) inhibitors, exhibiting strong antiproliferative activity and ROS-mediated cytotoxicity in human cancer cells, reinforcing the potential of organoselenium compounds in redox-targeted anticancer strategies [28]. Furthermore, corroborating the potential of selenium in cancer studies, researchers synthesized a selenium-containing TMZ analog (TMZ-Se). TMZ-Se demonstrated greater cytotoxicity than TMZ in glioma cells [29]. Additionally, several clinical trials have evaluated selenium-containing molecules, including the selenoamino acids selenomethionine and methylselenocysteine. Notably, these compounds are already being tested in later-phase clinical trials, including phase 3 for colorectal cancer (NCT00706121) and phase 4 for thyroid carcinoma (NCT04683575) [13]. Their progression to later-phase trials highlights their demonstrated efficacy and safety in preclinical and early phases of clinical studies.

Diphenyl diselenide (DPDS) is an organoselenium compound with well-documented pharmacological activities in different experimental models, including antitumor, cardioprotective, hepatoprotective, antioxidant, and anti-inflammatory effects [30–38]. However, its role in human glioblastoma remains poorly understood.

The literature suggests a strong link between oxidative stress and inflammation in cancer [39, 40], particularly in glioblastoma, where these processes contribute to tumor progression and therapy resistance [41, 42]. Considering the reported antioxidant and anti-inflammatory properties of DPDS, we hypothesized that DPDS, when co-exposed with temozolomide (TMZ), could modulate oxidative and inflammatory markers. Consequently, other pathways modulated by these processes may potentially reduce the pro-inflammatory profile of glioblastoma cells and increase the therapeutic efficacy of TMZ.

In this study, we investigated whether co-exposure of DPDS with TMZ could modulate oxidative stress and inflammation in glioblastoma cells (U87-MG), thereby potentially enhancing the therapeutic effects of TMZ. To this end, we analyzed outcomes related to cell viability and proliferation, as well as biochemical assays (ROS, CAT, and SOD) and molecular analyses, including markers of oxidative stress, endoplasmic reticulum stress, inflammation, apoptosis, hypoxia response, epigenetic regulators, cell cycle, DNA damage response, and epithelial-mesenchymal transition.

## Materials and Methods

### Chemicals

Diphenyl Diselenide, Temozolomide, 3-(4,5- dimethylthiazol-2-yl)−2,5-diphenyltetrazolium bromide (MTT), Sulforhodamine B (SRB), Dimethyl Sulphoxide (DMSO), 2′, 7′-dichlorofuoresceindiacetate (2, 7-DCFDA) and the other reagents were purchased from Sigma Aldrich (St Louis, MO, USA). The primers for the genes evaluated in the RT-qPCR assays in this study, TRIzol Reagent (Invitrogen, Carlsbad, CA), cDNA Reverse Transcription Kit (Applied Biosystems, Foster City, CA), and Power SYBR Green PCR Master Mix (Invitrogen) were purchased from Biogen (Cambridge, Massachusetts, USA). Dulbecco’s modified Eagle’s medium (DMEM) (Gibco^®^), Trypsin-EDTA (0,25%) (Gibco^®^), Fetal bovine serum (FBS) (Gibco^®^), and penicillin/streptomycin (Gibco^®^) were purchased from Biogen (Cambridge, Massachusetts, USA).

### U87-MG Cell Culture and Treatments

**Fig. 1:**
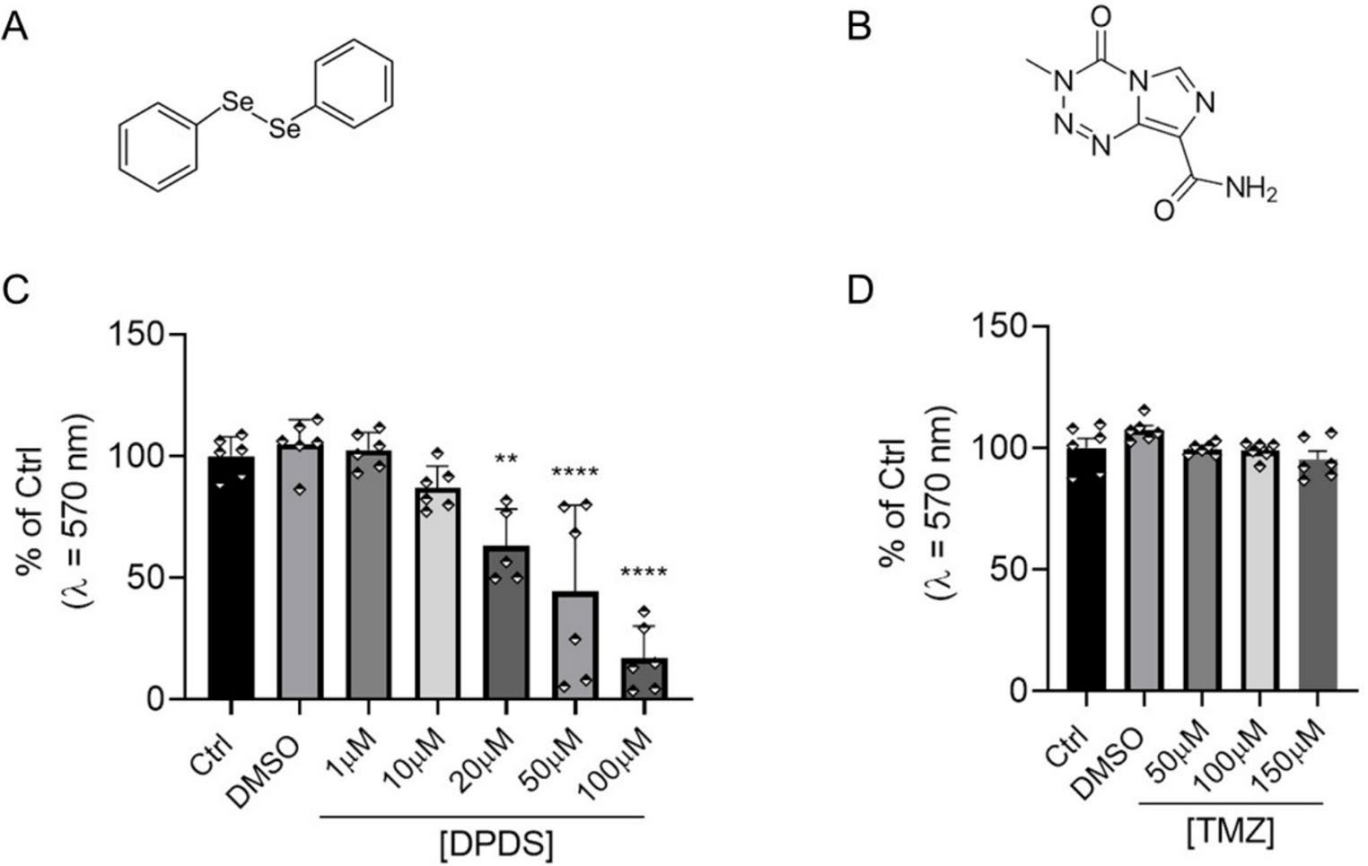
Effects of diphenyl diselenide (DPDS) and temozolomide (TMZ) on the viability of U87-MG glioblastoma cells. Chemical structures of (**A**) diphenyl diselenide and (**B**) temozolomide. Concentration curve of (**C**) diphenyl diselenide and (**D**) temozolomide through cell viability assay by MTT reduction. The data were expressed as mean ± SD and analyzed by one-way ANOVA followed by Tukey post hoc test. *Significantly different from the control group (*p < 0.05; **p < 0.01; ***p < 0.001; ***p < 0.0001)

**Fig. 2:**
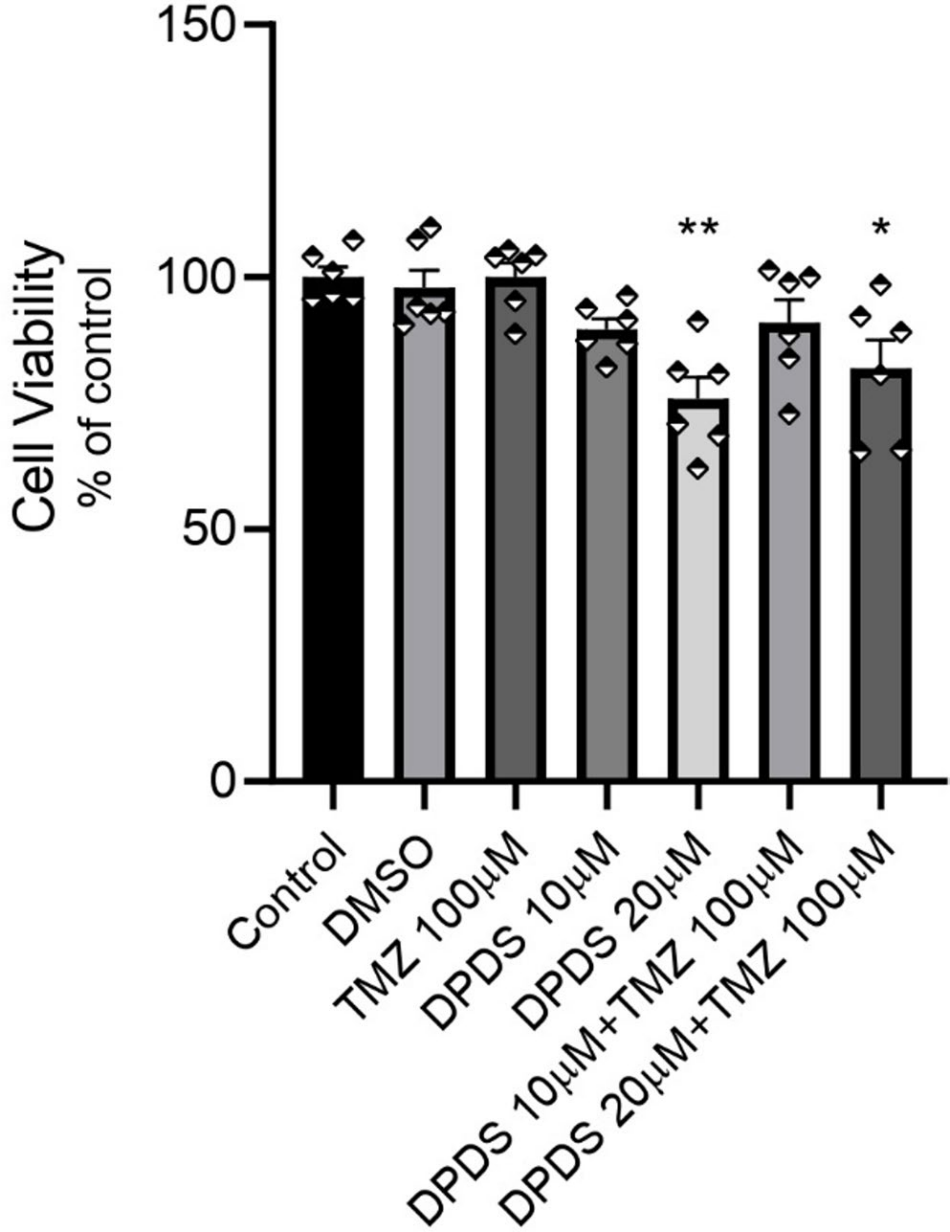
DPDS 20µM alone and in combination with TMZ 100µM decreased cellular MTT reduction. Data were expressed as mean ± SD and analyzed by one-way ANOVA followed by Tukey post hoc test. *Significantly different from the control group (*p < 0.05; **p < 0.01)

Human glioblastoma cells were purchased from the Rio de Janeiro Cell Bank (BCRJ) at passage 139. For assays, cells were cultured between passage 142–152 in DMEM (Dulbecco’s Modified Eagle’s medium; Gibco^®^) containing 1 g/L of D-glucose, L- Glutamine, 110 mg/ml sodium pyruvate and supplemented with 10% of heat inactivated (30 min, 56 ◦C) fetal bovine serum (FBS; Gibco^®^) and 1% penicillin/streptomycin (Gibco) in a humidified incubator at 37 ◦C and 5% CO2. DPDS and TMZ were prepared in DMSO. The concentrations of DPDS (20 µM) and TMZ (100 µM) were determined from concentration curves obtained in the MTT cell viability assay (Figs. 2 and 1). The doses were selected to investigate the potential adjuvant effect of the lowest concentration of DPDS capable of inducing cytotoxicity in tumor cells, in association with a concentration of TMZ that, alone, does not have a significant cytotoxic effect on these cells.

Regarding the DPDS concentration used in this study, cell viability, density, and migration assays indicated that 20 µM represents an appropriate condition for the initial evaluation of its anticancer capacity. This concentration has previously been reported as non-toxic in in vivo models, such as *Drosophila melanogaster* [34, 43, 44], as well as in human lymphocytes (PBMCs), where no cytotoxicity effects were observed [35]. Additionally, other reports have shown that DPDS at a concentration of 100 µM does not reduce cell viability in primary cultures of murine astrocytes. In contrast, it causes loss of cell viability at 10 µM in C6 cells (glioma of murine origin) [45].

For all concentrations evaluated in this study, stock solutions were prepared in advance to prevent changes in the composition of the culture medium (DMEM). The different treatment concentrations were standardized, as was the vehicle control group (DMSO), whose proportion was kept constant at 0.2% of the final volume of each well in the cell culture plates.

### Cell Viability by MTT Assay

Determination of cellular toxicity of the compounds in U87-MG was performed by MTT assay, following the methodology described by [46], with some modifications. The method is based on the enzymatic reduction of MTT (3-(4,5-dimethylthiazol2-yl)−2,5-diphenyl tetrazolium bromide) to formazan crystals by mitochondrial dehydrogenases. Glioblastoma Cells (5 × 10^3^) were seeded in 96-well plates (Kasvi^®^) and incubated at 37 ◦C for 48 h to allow adequate adhesion to the plates (confluence between 70% and 90%) according to [20] and [47] with minor modifications. After that, treatment was performed for 24 h with DPDS and TMZ in different concentrations. DPDS effects were evaluated at the following concentrations: 1 µM, 10 µM, 20 µM, 50 µM, and 100 µM. TMZ effects were assessed at the following concentrations: 50 µM, 100 µM, and 150 µM. After treatment, MTT was added to the medium, and the samples were incubated for 4 h at 37 °C, in the dark. Subsequently, the samples were centrifuged, the supernatant was discarded, and the formazan crystals were solubilized with DMSO p.a. After, the samples were readied at 540 nm in a SpectraMax M5 reader. Results were expressed as % of the control.

### Sulforhodamine B (SRB) Cell Mass Assay

To evaluate cell density, the SRB assay based on [48] was performed, with minor modifications. Briefly, similar to the MTT assay, glioblastoma cells (5 × 10^3^) were seeded in 96-well plates (Kasvi^®^) and incubated at 37 ^◦^C for 48 h to allow adequate adhesion to the plates (confluence between 70% and 90%). Through the MTT assay, we determined that two low concentrations of DPDS (10 µM and 20 µM) and one high concentration of TMZ (100 µM) would be used to continue the coexposure analyses of these compounds in glioblastoma cells. We determined a non-cytotoxic concentration of TMZ (100 µM) based on our results to evaluate the potential of co-exposure with DPDS. The literature supports our findings, showing that TMZ begins to reduce the viability of U87-MG cells only up to 100 µM in 24 h of exposure [20, 49–51]. Cell masses were determined based on the SRB cell protein stain. Cells were fixed (10% trichloroacetic acid, 4 °C, 1 h), water washed, dried, stained (100 µl 0.4% SRB in 1% acetic acid, 30 min at RT), and then washed four times (1% acetic acid). Dissolved SRB (10 mM Tris, pH 10) was quantified (564 nm, SpectraMax M5 reader).

### Migration Assay

The cell migration assay was performed according to [20], [20], with some minor modifications. Cells were grown in 6-well plates (Kasvi) until they reached 80–90% confluence. Then, a 10 µL pipette tip was used to create a fine scratch (wound) in the central area of the well. The wells were washed three times with PBS to remove any detached or damaged cells, and the medium with the different treatments was added. DPDS (10 µM and 20 µM) and TMZ (100 µM), individually or in combination, were added, and migration to the open area was documented at time zero and after 24 h. Images were obtained with an Evos XL Core inverted microscope by Invitrogen (Thermo Fischer Scientific). Wound closure was quantified using ImageJ software to measure the distance from the wound edges (area), and the results are presented as the percentage of wound closure relative to untreated cells (Vehicle-DMSO Group).

### Biochemical Assays

#### Preparation of Total Cell Lysates

Total cell lysis was performed according to the method described by [20, 23], with some minor modifications. Briefly, cells were grown in 6-well plates (Kasvi^®^) until they reached 80–90% confluence. After the expected confluence, we removed the old medium and added new medium with the treatments. Based on previous assays, we decided to continue with only the following groups for subsequent assays: (1) Control – DMSO vehicle, (2) 20 μm DPDS, (3) 100 μm TMZ, and (4) 20 μm DPDS + 100 μm TMZ. After 24 h of exposure, the treatment media were removed, and the cells were washed with 1× phosphate-buffered saline (PBS). After washing, to avoid potential interference from Triton and other lysis buffers in biochemical techniques, we added 300 µl of 1X PBS to each well. We mechanically scraped the cells with a cell scraper to promote cell lysis. After this, the content originating from the scraping of each well was transferred to a microtube. The samples were lysed again by moving them “up and down” in a 1 mL insulin syringe. The samples were then centrifuged (3000 rpm/10 min/4 °C), and the supernatant was separated for protein measurement and biochemical tests.

#### Protein Quantification

Sample protein quantification was measured by the Lowry method [52] for all the parameters. The color reagent used was the Folin–Ciocalteu. Bovine serum albumin was used for the standard curve.

#### Reactive Oxygen Species Assay

Reactive oxygen species (ROS) production in the U87-MG cell supernatant was determined using a technique based on the oxidation of the 2′7′ dichlorofluorescein diacetate (H2DCF-DA) described by LeBel and colleagues et al., 1992 [53]. The samples were incubated in a medium containing 100 µM H2DCF-DA. Then, the reaction produces a fluorescent compound, dichlorofluorescein (DCF), which is determined at an excitation wavelength of 488 nm and an emission wavelength of 525 nm. The data were plotted as nM DCF per mg of protein.

#### Catalase Activity Assay

Briefly, the supernatants were mixed with 0.1% Triton X-100, preincubated for 15 min at room temperature, and then 10 mM potassium phosphate buffer (pH 7.0) with 20 mM H_2_O_2_ as previously described by [54]. A blank was prepared with buffer and H_2_O_2_. A CAT unit is defined as 1 mM H_2_O_2_ consumed per minute at 37 ◦C. H_2_O_2_ degradation was measured at 240 nm. The activity was expressed in units per mg of protein.

#### Superoxide Dismutase Activity Assay

To determine superoxide dismutase (SOD) activity, we used the Marklund method [55]. The inhibition of pyrogallol autoxidation was measured at 420 nm and was indirectly associated with SOD activity in the samples. A calibration curve was performed using purified SOD as the standard, and the results were expressed as units per mg of protein.

### RNA Extraction and RT-qPCR

Total RNA was isolated from GBM cultures using TRIzol Reagent according to the manufacturer’s instructions (Invitrogen, Carlsbad, CA). The concentration and purity of the RNA were spectrophotometrically determined at a ratio of 260/280. Subsequently, 1 µg of total RNA was reverse transcribed using the Applied Biosystems™ High-Capacity cDNA Reverse Transcription Kit (Applied Biosystems, Foster City, CA) in a 20 µL reaction, according to the manufacturer’s instructions. The mRNA quantification of NRF2, CAT, SOD, NFκB1, HDAC4, BAX, BAD, BCL-2, GPX1, GPX4, SNAI1, PTGS2, HIF1A, SELENOS, DIO2, IL6, IL-1B, TNFα, NEK1 and NEK2 was performed using primer pairs (Table 1) and Power SYBR Green PCR Master Mix (Invitrogen). Quantitative RT-PCR was performed in duplicate using the Applied Biosystems 7500 Fast system. No-template and no-reverse transcriptase controls were included in each assay, producing no detectable signal during the 35–40 cycles of amplification. Target mRNA levels were normalized to β-actin levels using the 2-ΔΔCt method [56, 57].

**Table 1:** Oligonucleotide primers for real-time RT-PCR. NRF2 (NFE2L2) – Nuclear factor erythroid 2–related factor 2; KEAP1 - Kelch-like ECH-associated protein 1; TXNRD1- Thioredoxin Reductase 1; TXNRD2- Thioredoxin Reductase 2; NFKB1 – Nuclear factor kappa B subunit 1; HDAC4 – Histone deacetylase 4; BAX – Bcl-2–associated X protein; BAD – Bcl-2–associated death promoter; BCL2 – B-cell lymphoma 2; GPX4 – Glutationa peroxidase 4; SNAI1 – Snail family transcriptional repressor 1; PTGS2 (COX-2) – Prostaglandin-endoperoxide synthase 2; HIF1A – Hypoxia-inducible factor 1 alpha; SELENOS – Selenoprotein S; DIO2 – Deiodinase, iodothyronine type II; ACTB – Beta-actin; NEK1 – NIMA related kinase 1; NEK2 – NIMA related kinase 2; IL6 – Interleukin 6; IL1B (IL-1β) – Interleukin 1 beta; TNFα (TNF) – Tumor necrosis factor alpha; GPX1 – Glutationa peroxidase 1

Mrna Target	Forward (5’−3’)/Reverse (5’−3’)
NRF2	AGGTTGCCCACATTCCCAAA AACGTAGCCGAAGAAACCTCA
KEAP1	CCACAACAGTGTGGAGAGGTAT CCACGGCATAAAGGAGACGA
TXNRD1	ATTTGCAGCAGAGCGAAAGG GCCCGACCGTCCTAAGAAT
TXNRD2	GATCCAAGATGCCCCCAACT TCTGCCATCTTCCTCCAGTCA
NFKB1	ATGGGCTACACCGAAGCAAT TCTCGGAGCTCGTCTATTTGC
HDAC4	CGTCAACATGGCTTTCACCG ATGACCACCGTTCTGAAGGC
BAX	CCCTTTTGCTTCAGGGTTTCAT ACTCGCTCAGCTTCTTGGTG
BAD	TTGTGGACTCCTTTAAGAAGGGAC AAGTTCCGATCCCACCAGGA
BCL2	GATGGGATCGTTGCCTTATGC CAGTCTACTTCCTCTGTGATGTTGT
GPX4	AGAGATCAAAGAGTTCGCCGC TCCACTTGATGGCATTTCCCAG
SNAI1	GTTCTTCTGCGCTACTGCTG TGCTGGAAGGTAAACTCTGGATT
PTGS2	CCACCCGCAGTACAGAAAGT TGCAGACATTTCCTTTTCTCCTGT
HIF1A	CGCGAACGACAAGAAAAAGATAAG GTGGCAACTGATGAGCAAGC
SELENOS	GTCTTTCAGAAGCTTTCCGCC CAACAACATCAGGTTCCACAGC
SELENOP	CTGCTCTCTCACGACTCTCAA GAAAGCTCACTGCTGCCAAG
SELENOI	TGACTTTTATGCCTCAGCACCA ACGAAGTTGAGGATGCCCAC
SELENOK	GCTGGTGGATGAGGAAGGTAAAT CATGGTCAGCCTTCCACTTCT
DIO2	CGATGCCTACAAACAGGTGAAA TTCTCCTGGGTACCATTGCC
ACTB	GCCGAGACCGCGTCC ATCATCCATGGTGAGCTGGC
NEK1	GGAGGGGATCTGTTTAAGCGAA ACCAGTCCAAAATCTGATCCTCTTG
NEK2	AGGGAACCAAGGAAAGGCAA CAGGGCCAGAGTCAACTGAG
IL6	GCCCAGCTATGAACTCCTTCT CTTCTCCTGGGGGTACTGG
IL-1B	TACCTGTCCTGCGTGTTGAA TCTTTGGGTAATTTTTGGGATCT
TNFα	TGCACTTTGGAGTGATCGGC AGCTTGAGGGTTTGCTACAACA
GPX1	TATCGAGAATGTGGCGTCCC TCTTGGCGTTCTCCTGATGC

### Statistical Analyses

All data were expressed as mean ± standard deviation (SD). Experiments were performed at least in triplicate (sample size equal to or greater than 3). Statistical analyses were performed using GraphPad Prism8 Software. All performances were assessed by one-way or two-way analysis of variance (ANOVA), followed by the Tukey post hoc test. Differences were considered statistically significant among groups when *p* < 0.05.

## Results

### Cell Viability and Determination of DPDS and TMZ Concentrations

First, we generated concentration curves (Fig. 2) to determine the optimal concentrations for subsequent analyses of DPDS and TMZ. DPDS caused a reduction in cell viability from 20 to 100 µM (Fig. 2C), whereas TMZ did not affect cell viability at the concentrations evaluated (50, 100, and 150 µM; Fig. 1D). This finding is in agreement with literature data, which show that TMZ is effective only at concentrations above 250 µM after treatment for 24-hour [20, 49, 50]. We worked with non-toxic concentrations of TMZ because the focus of this study was to assess the possible potentiation of DPDS toxicity by a non-effective concentration of TMZ (100 µM) in GBM. We selected two concentrations of DPDS: the highest that did not promote cytotoxicity (DPDS 10 µM) and the lowest that caused some damage (DPDS 20 µM), and performed co-exposure with 100 µM TMZ. Only the combined exposure of DPDS 20 µM and TMZ significantly reduced the viability of GBM cells (*p* < 0.05) compared to the vehicle control group (DMSO) (Fig. 2).

### DPDS decreases GBM Cell Density and Potentiates TMZ Cytotoxicity in GBM Cells

**Fig. 3:**
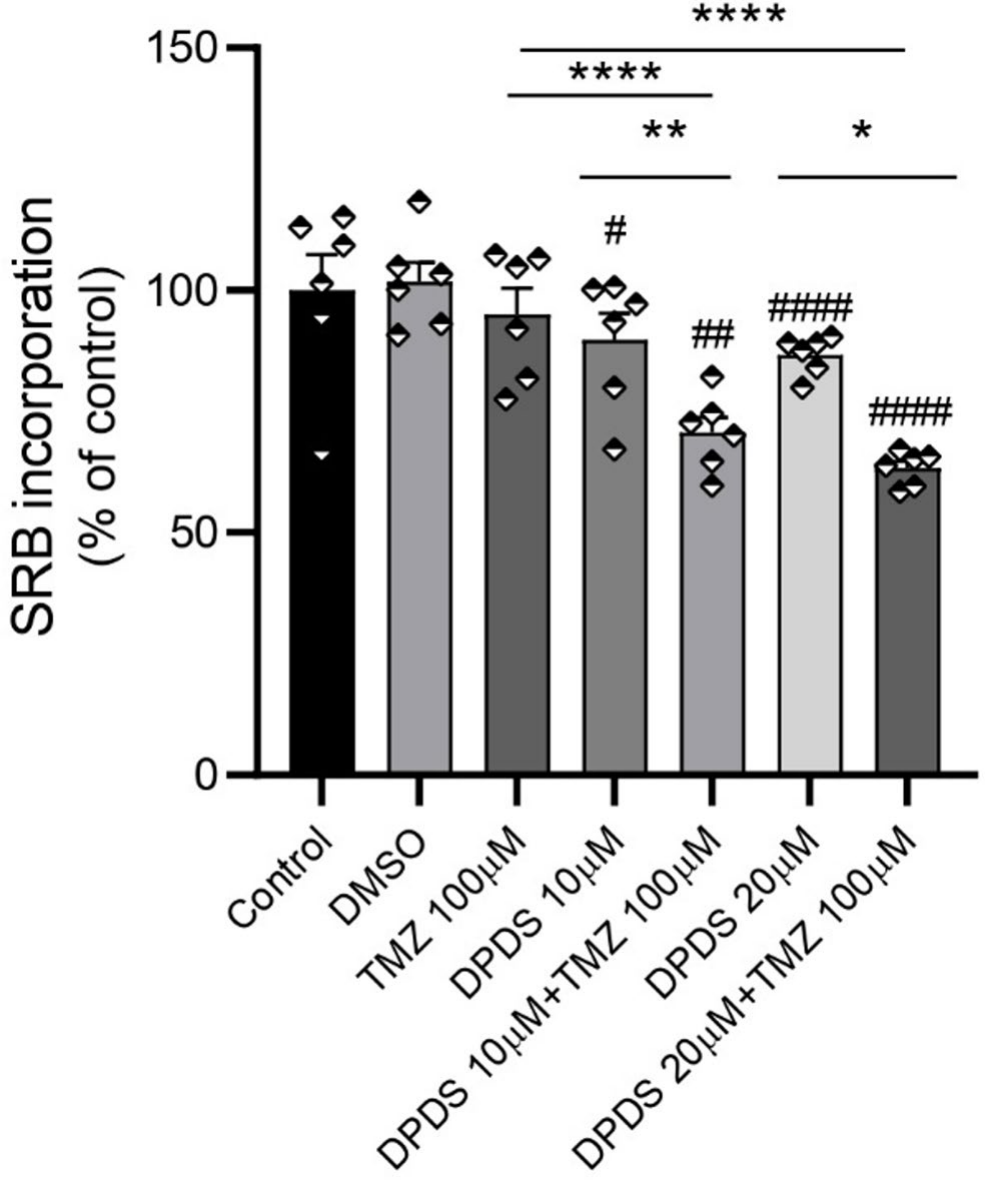
DPDS reduced cell density. Co-exposure of DPDS and TMZ potentiated the reduction in cell density. # represents a statistical difference compared to the vehicle group (^#^*p* < 0.05; ^##^*p* < 0.01; ^###^*p* < 0.001; ^####^*p* < 0.0001). * represents a difference between two distinct groups (*p < 0.05; **p < 0.01; ***p < 0.001; ****p < 0.0001). The data were expressed as mean ± SD and analyzed by one-way ANOVA followed by Tukey post hoc test

DPDS reduced GBM cell density as the concentration increased from 10 µM to 20 µM. TMZ did not reduce cell density after 24 h of incubation. In co-exposure, a potentiation effect was observed, with DPDS + TMZ significantly reducing GBM cell density compared to the DMSO, TMZ, DPDS 10 µM, and DPDS 20 µM groups, indicating a pro-cytotoxic interaction between DPDS and TMZ in GBM (Fig. 3).

### DPDS, TMZ, and Their Combination Reduce the Cellular Migration of GMB Cells

**Fig. 4:**
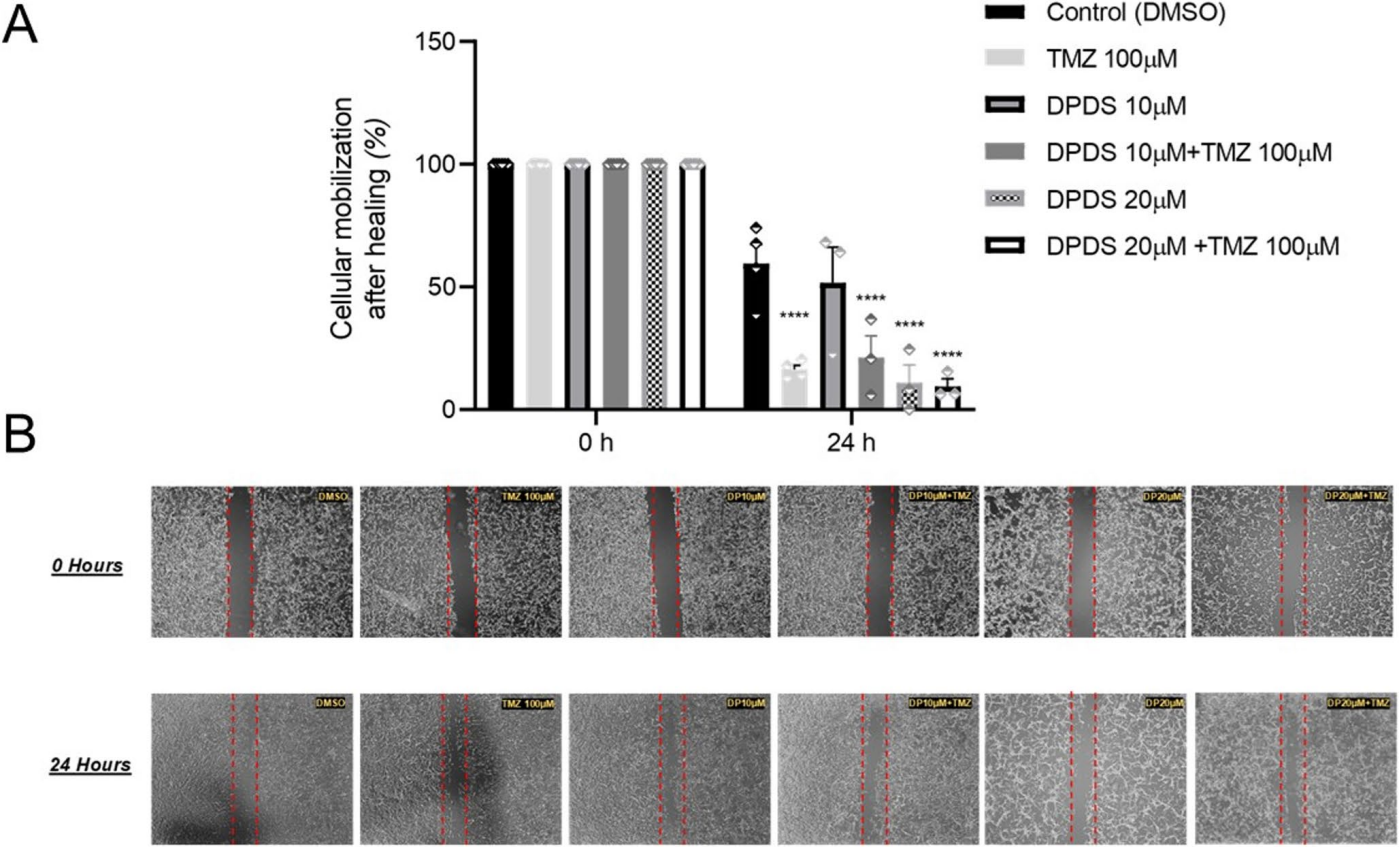
DPDS impaired cell mobilization of U87-MG glioblastoma. (A) Treatment with DPDS (10–20 μM), alone or in co-exposure with TMZ (100 μM), significantly decreased cell mobilization in 24 h in the wound healing assay. (B) Representative image of the technique. The data were expressed as mean±SD and analyzed by two-way ANOVA followed by Tukey’s post hoc test. *Significantly different from the control group (****p<0.0001)

In the migration assay (Fig. 4), Treatment with 20 µM of DPDS significantly reduced cell migration. Groups treated with TMZ and simultaneously with DPDS also showed reduced cell migration. For the biochemical and molecular analyses described below only the 20 µM DPDS concentration was used, which resulted in four experimental groups: (1) Control – DMSO vehicle, (2) 20 µM DPDS, (3) 100 µM TMZ, and (4) 20 µM DPDS + 100 µM TMZ.

### Co-Exposure of DPDS and TMZ Promoted Alteration in Oxidative Stress Parameters in GBM Cells

**Fig. 5:**
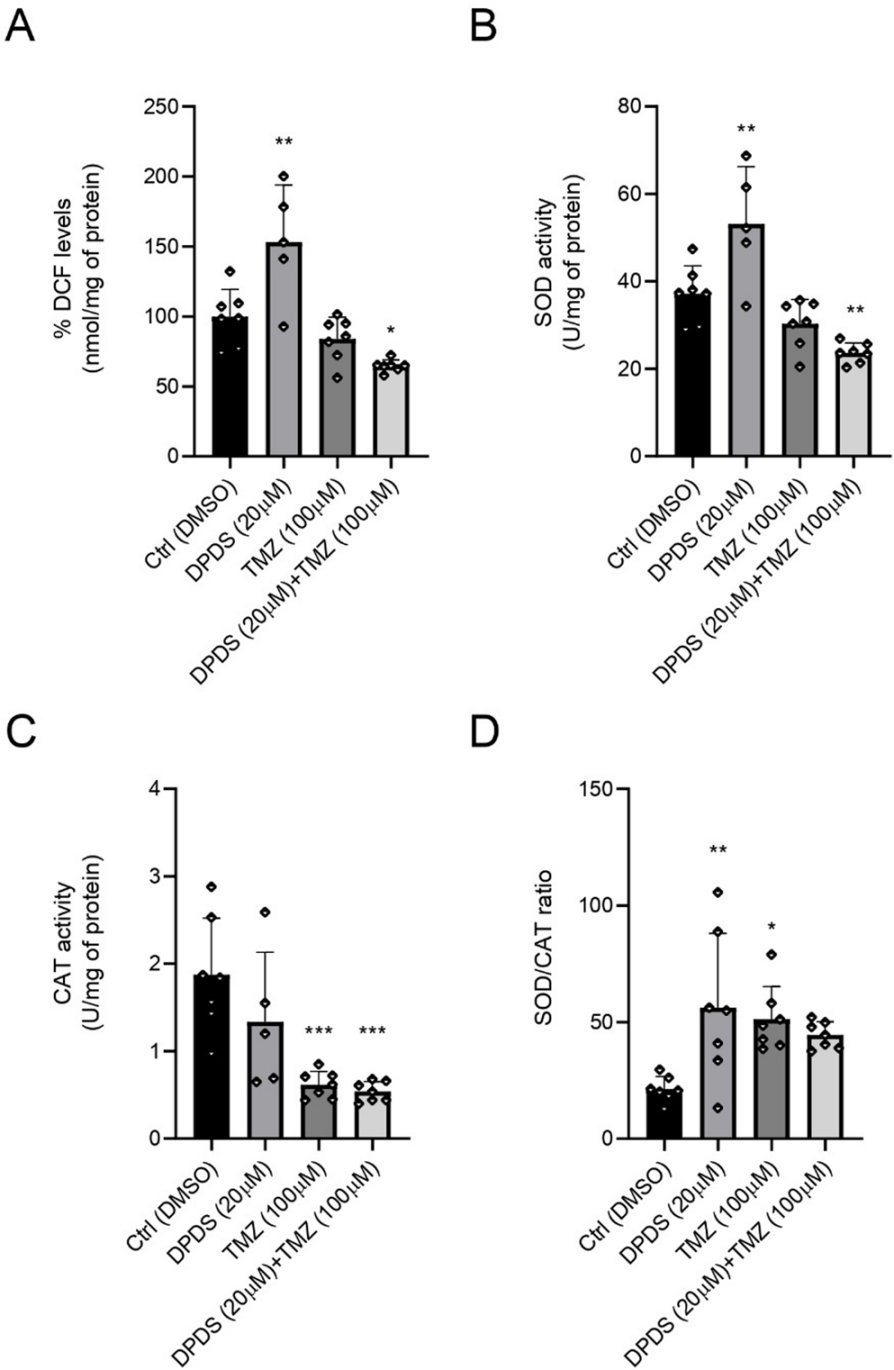
Effects of DPDS and TMZ on oxidative stress markers in U87-MG cells. Cells were treated with DPDS (20 µM), TMZ, or their combination. (**A**) Intracellular reactive oxygen species (ROS) levels. (**B**) Superoxide dismutase (SOD) activity. (**C**) Catalase (CAT) activity. (**D**) SOD/CAT ratio. DPDS increased ROS and SOD activity, whereas TMZ alone had minimal effects on these enzymes. Co-treatment reduced ROS and antioxidant enzyme activities, indicating modulation of redox balance in GBM cells. The data were expressed as mean ± SD and analyzed by one-way ANOVA followed by Tukey’s post hoc test. *Significantly different from the control group (*p < 0.05; **p < 0.01; ***p < 0.001)

Treatment with DPDS alone promoted an increase in reactive oxygen species (DFCH oxidation; *p* < 0.01; Fig. 5A) and SOD enzyme activity (*p* < 0.01; Fig. 5B), but did not alter CAT enzyme activity compared to the control group (Fig. 5C). In contrast, the co-exposure to DPDS and TMZ caused a significant decrease in reactive oxygen species production (*p* < 0.05). Meanwhile, treatment with TMZ alone significantly reduced CAT enzyme activity compared to the control group (*p* < 0.001; Fig. 5C). The ratio between the enzymatic activities of SOD and CAT was calculated, and the groups treated alone with DPDS (*p* < 0.01) and TMZ (*p* < 0.05) exhibited a significant increase in the SOD-to-CAT ratio compared to the control group (Fig. 5D). The group exposed simultaneously to DPDS + TMZ exhibited a significant reduction in the enzymatic activities of SOD (*p* < 0.01; Fig. 5B) and CAT (*p* < 0.001; Fig. 5C).

### Transcriptional Suppression of Oxidative Stress genes induced by DPDS and TMZ

**Fig. 6:**
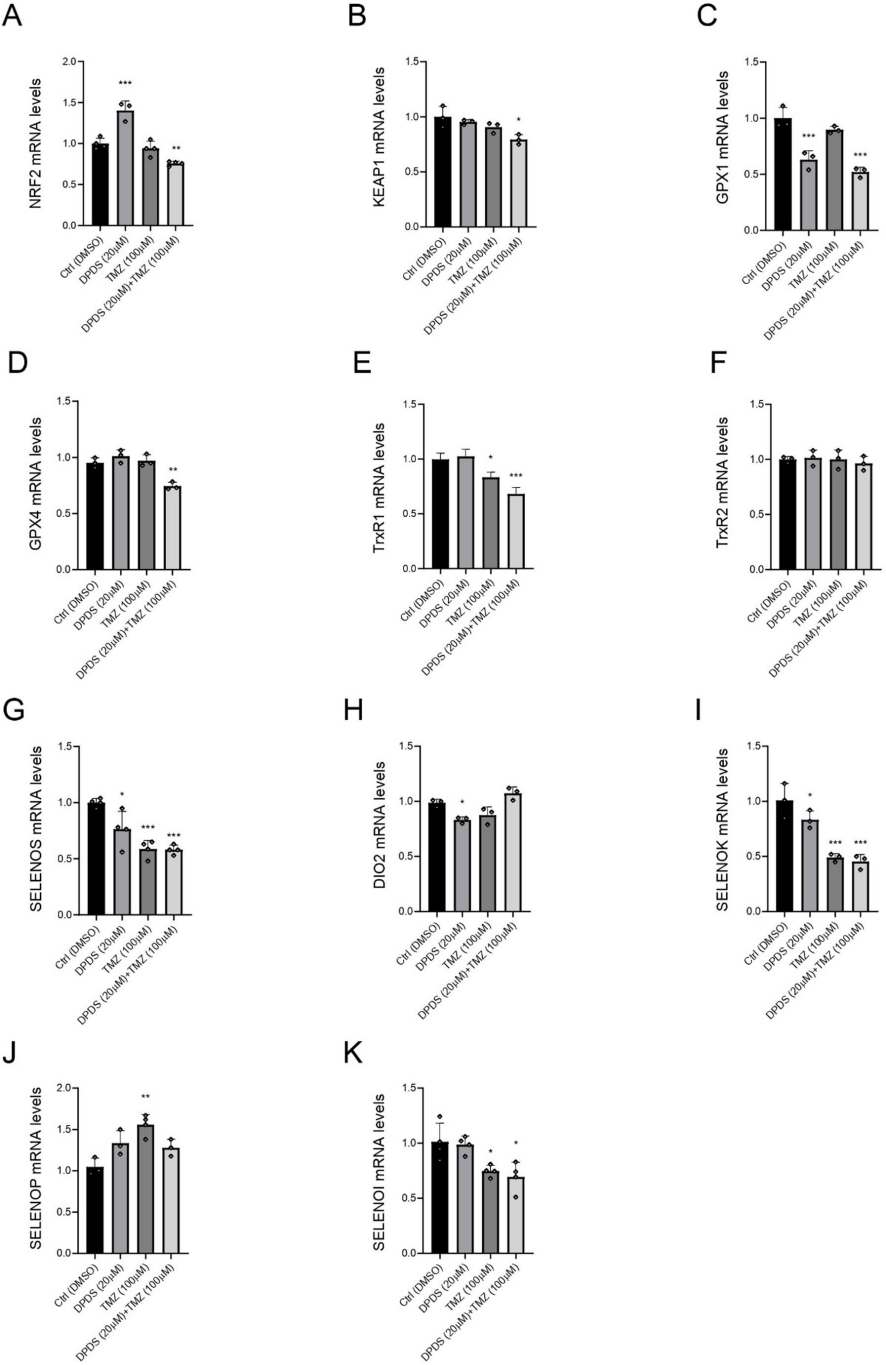
DPDS and TMZ modulate gene expression of redox-related proteins in U87-MG cells. Relative mRNA levels of (**A**) NRF2; (**B**) KEAP1; (**C**) GPX1; (**D**) GPX4; (**E**) TXNRD1; (**F**) TXNRD2; (**G**) SELENOS; (**H**) DIO2; (**I**) SELENOK; (**J**) SELENOP; (**K**) SELENOI. Data were expressed as mean ± SD and analyzed by one-way ANOVA followed by Tukey’s post hoc test. *Significantly different from the control group (*p < 0.05; **p < 0.01; ***p < 0.001; ***p < 0.0001)

GBM cells treated with DPDS alone showed increased gene expression of NRF2 (*p* < 0.001; Fig. 6A), and reduced gene expression of GPX1 (*p* < 0.001; Fig. 6C). Meanwhile, treatment with TMZ reduced gene expression of TXNRD1 (*p* < 0.05; Fig. 6E) and increased SELENOP mRNA levels (*p* < 0.01; Fig. 6J). Co-exposure to DPDS and TMZ reduced gene expression of NRF2 (*p* < 0.01; Fig. 6A), KEAP1 (Fig. 6B; *p* < 0.05), GPX1 (*p* < 0.001; Fig. 6C), GPX4 (*p* < 0.01; Fig. 6D), TRXR1 (*p* < 0.001; Fig. 6E), and SELENOI (*p* < 0.05; Fig. 6K)). Treatments did not alter TXNRD2 expression (Fig. 6F).

### Downregulation of Endoplasmic Reticulum Stress Genes Induced by DPDS and Co-Exposure with TMZ

Treatment with DPDS alone reduced the gene expression of DIO2 (*p* < 0.05; Fig. 6H), SELENOS (*p* < 0.05; Fig. 6G), SELENOK (*p* < 0.05; Fig. 6I), and SELENOI (*p* < 0.05, Fig. 6K), while treatment with TMZ alone, as in co-exposure, reduced the gene expression of SELENOS (*p* < 0.001; Fig. 6G) and SELENOK (*p* < 0.001; Fig. 6I).

### DPDS, TMZ, and Their Co-Exposure Suppress Pro-Inflammatory Gene Expression

**Fig. 7:**
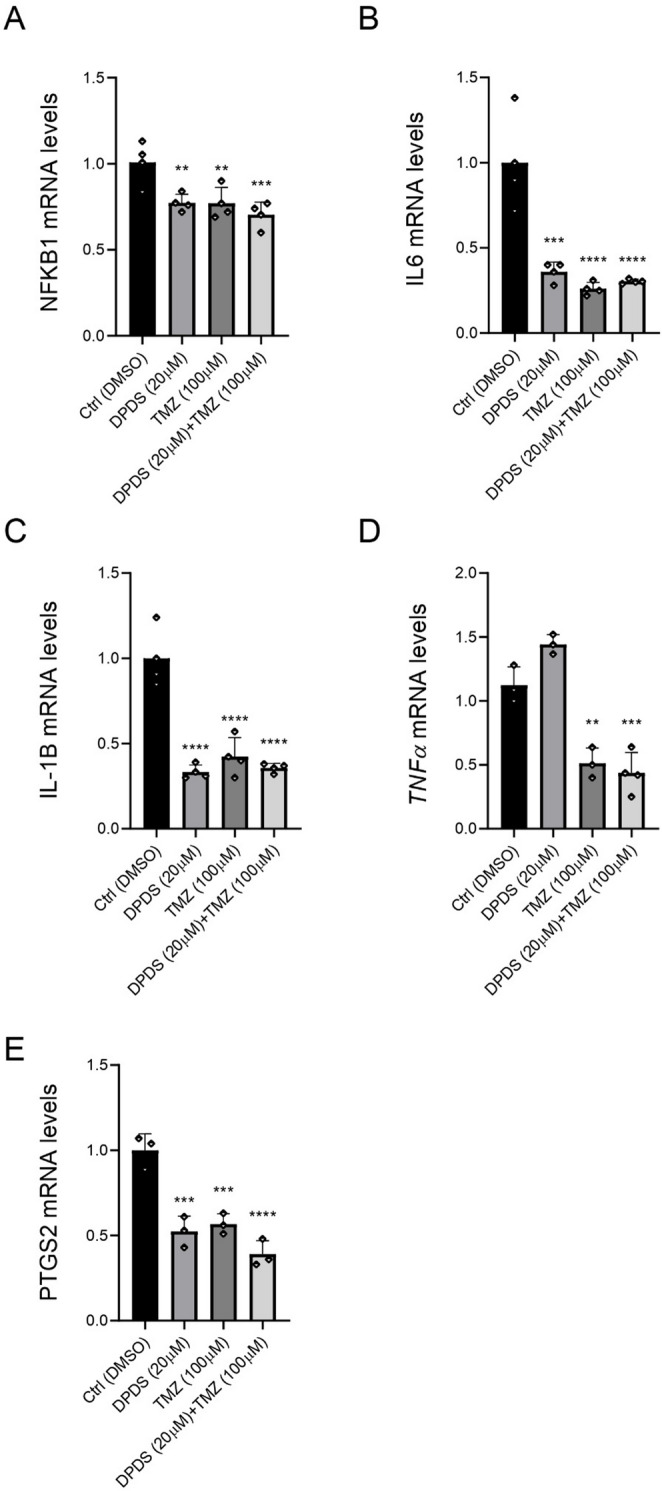
DPDS and TMZ modulate gene expression of inflammatory markers in U87-MG cells. Relative mRNA levels of (**A**) NFKB1; (**B**) IL6; (**C**) IL-1B; (**D**) TNFα; (**E**) PTGS2. The data were expressed as mean ± SD and analyzed by one-way ANOVA followed by Tukey’s post hoc test. *Significantly different from the control group (*p < 0.05; **p < 0.01; ***p < 0.001; ***p < 0.0001)

The isolated treatments with DPDS and TMZ, and their combination, efficiently decreased the gene expression of various pro-inflammatory genes. DPDS decreased NFKB1 (*p* < 0.01; Fig. 7A), IL6 (*p* < 0.001; Fig. 7B), IL-1B (*p* < 0.0001; Fig. 7C) and PTGS2 (*p* < 0.001; Fig. 7E) mRNA expression; and TMZ caused a reduction in NFKB1 (*p* < 0.01, Fig. 7A), IL6 (*p* < 0.0001; Fig. 7B), IL-1B (*p* < 0.0001; Fig. 7C), TNFα (*p* < 0.01; Fig. 7D) and PTGS2 (*p* < 0.001, Fig. 7E). The co-exposure to TMZ and DPDS decreased the expression of NFKB1 (*p* < 0.001; Fig. 7A), IL6 (*p* < 0.0001; Fig. 7B), IL-1B (*p* < 0.0001; Fig. 7C), TNFα (*p* < 0.001; Fig. 7D), and PTGS2 mRNA (*p* < 0.0001; Fig. 7E).

### Co-Exposure to DPDS and TMZ Downregulates Apoptosis-Related Genes

**Fig. 8:**
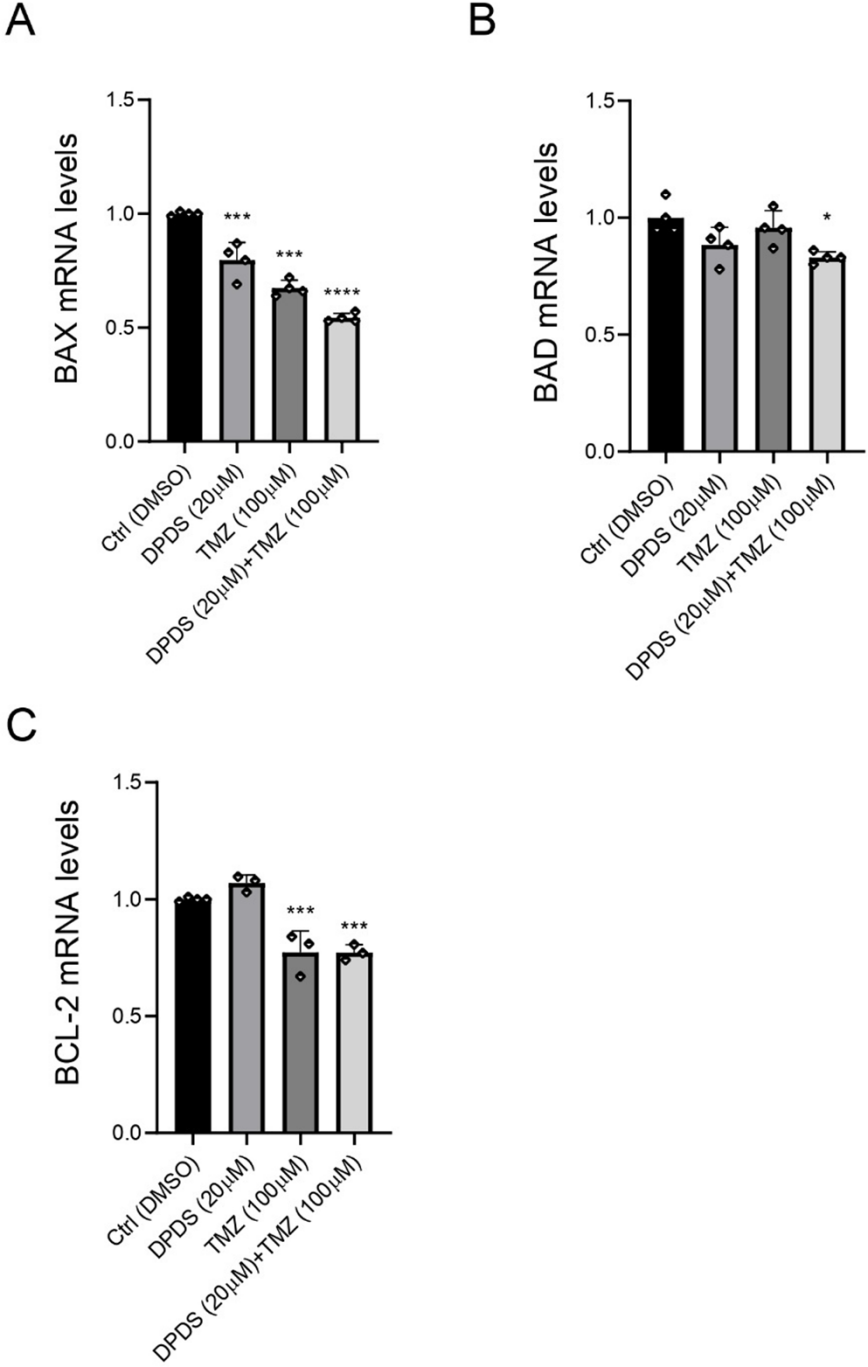
DPDS and TMZ modulate gene expression of apoptosis markers in U87-MG cells. Relative mRNA levels of (**A**) BAX; (**B**) BAD; (**C**) BCL-2. The data were expressed as mean ± SD and analyzed by one-way ANOVA followed by Tukey’s post hoc test. *Significantly different from the control group (*p < 0.05; ***p < 0.001; ***p < 0.0001)

DPDS alone reduced BAX mRNA expression (*p* < 0.001; Fig. 8A), while TMZ alone reduced BAX (*p* < 0.001; Fig. 8A) and BCL-2 (*p* < 0.001; Fig. 8C) mRNA expression. DPDS and TMZ co-exposure resulted in a reduction in BAX (*p* < 0.0001; Fig. 8A), BAD (*p* < 0.05; Fig. 8B), and BCL-2 mRNA expression (*p* < 0.001; Fig. 8C).

### Co-Exposure to DPDS and TMZ Downregulates Hypoxia Response and Epigenetic Regulators

**Fig. 9:**
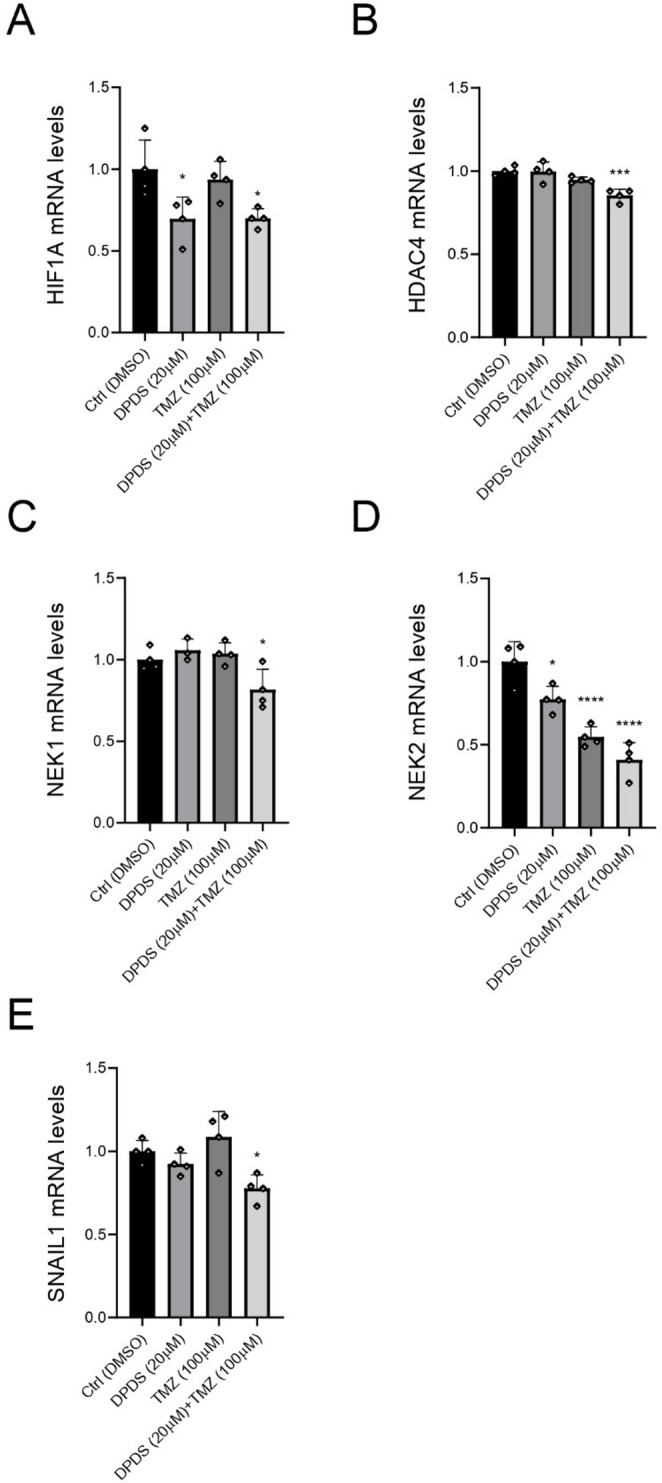
DPDS and TMZ modulate gene expression of hypoxia response, epigenetic regulators, cell cycle, DNA damage response and Epithelial–Mesenchymal Transition (EMT) markers in U87-MG cells. Relative mRNA levels of (**A**) HIF1A; (**B**) HDAC4; (**C**) NEK1; (**D**) NEK2; (**E**) SNAIL1. The data were expressed as mean ± SD and analyzed by one-way ANOVA followed by Tukey’s post hoc test. *Significantly different from the control group (*p < 0.05; ***p < 0.001; ****p < 0.0001)

DPDS alone reduced the expression of HIF1A mRNA (*p* < 0.05; Fig. 9A). Co-exposure to DPDS and TMZ decreased the HIF1A (*p* < 0.05; Fig. 9A) and HDAC4 mRNA expression (*p* < 0.001; Fig. 9B). TMZ alone did not affect the gene expression of HIF1A (Fig. 9A) and HDAC4 (Fig. 9B).

3.10 DPDS and TMZ Suppress the Expression of Key Regulators of Cell Cycle, DNA Damage Response, and Epithelial–Mesenchymal Transition (EMT).

GBM cells treated with DPDS or TMZ alone exhibited a reduction in gene expression only for NEK2 (*p* < 0.05 and *p* < 0.0001, respectively; Fig. 9D). The effects were more pronounced in co-exposure to DPDS and TMZ, with a significant decrease in gene expression of NEK1 (*p* < 0.05; Fig. 9C), NEK2 (*p* < 0.0001; Fig. 9D), and SNAI1 mRNA (*p* < 0.05; Fig. 9E).

## Discussion

GBM is characterized by rapid growth and invasiveness, which pose significant therapeutic challenges [58, 59]. The standard treatment for GBM involves surgical resection followed by radiotherapy and temozolomide (TMZ) chemotherapy [60]. Although TMZ is an effective DNA-alkylating agent, its efficacy is limited by tumor heterogeneity, genetic mutations, and resistance mechanisms, which contribute to poor outcomes [8, 12, 59, 61, 62]. Due to those constraints, there is a need to seek new therapeutic strategies to improve patient survival, such as identifying new drug targets for GBM, as well as new compounds capable of enhancing or replacing standard treatment with TMZ.

Our new findings indicate that DPDS reduces cell viability, density, and migration of GBM cells (Figs. 2, 1, 3 and 4), in addition to modulating several pathways associated with the antioxidant response, oxidative stress, inflammation, apoptosis, hypoxia response, and cell cycle (Figs. 5, 6, 7, 8 and 9). These effects were even more pronounced when DPDS was combined with TMZ.

Furthermore, it is important to highlight that pharmacokinetic studies described in the literature demonstrate that in patients with GBM, the maximum plasma concentration of TMZ ranges from approximately 50 to 100 µM [63, 64]. Indeed, the inhibitory effects of DPDS against glioma described in this work agree with a previous publication indicating that DPDS was able to reduce cell viability at 10 µM [45]. Furthermore, DPDS and derivatives have been reported to exhibit anticancer effects in other cancer cell lines [65–67].

Several studies have highlighted that the tumor microenvironment is characterized by an immunosuppressive profile, often associated with high levels of oxidative stress and inflammation [40, 42, 68]. Notably, selenoprotein K (SELENOK) plays an important role in immune cell activation and calcium-dependent signaling [69, 70], and alterations in its expression may interfere with tumor immune evasion. In this context, molecules capable of modulating these pathways, such as DPDS, emerge as promising candidates for adjuvant strategies aimed at reducing the pro-tumor environment.

Our findings demonstrated that the combination of DPDS and TMZ reduced ROS production and the activity of SOD and CAT enzymes. Additionally, DPDS alone increased ROS production and SOD activity (Fig. 5). In a similar way to that reported for sodium selenite, Kim and collaborators [71] demonstrated that superoxide anion generated by selenite triggered mitochondrial damage, subsequent mitophagy and cell death in glioma cells.

Overall, our results indicated that DPDS modulated the inflammatory response (as determined by a decrease in the expression of IL6, IL1B, NFKB1, and PTGS2) and the redox response (e.g., GPX1, DIO2, SELENOK, and SELENOS) negatively. In combination with TMZ, DPDS further reinforced these effects, leading to a broader suppression of inflammatory mediators (IL6, IL1B, NFKB1, TNFα, and PTGS2) and additional downregulation of oxidative stress–related genes (NRF2, GPX1, GPX4, SELENOS, KEAP1, SELENOK, SELENOI, and TRXR1) (Figs. 6 and 7).

Our observations align with those of Wang and colleagues, who demonstrated that DPDS exhibits both antioxidant and anti-inflammatory properties both in vitro and in vivo, thereby reinforcing its potential in conditions associated with oxidative stress [72]. These findings connect with the results of Mancini and colleagues, who demonstrated the ability of DPDS to promote the activation and nuclear translocation of Nrf-2 factor in macrophage cells [73].

In normal cells, antioxidant pathways act as cytoprotective signaling, maintaining redox homeostasis and promoting survival under transient oxidative stress [74, 75]. In contrast, cancer cells undergo malignant redox reprogramming marked by the overproduction of reactive oxygen species (ROS) and chronic oxidative stress that sustains tumor growth [76, 77]. To cope with this condition, tumor cells become highly dependent on hyperactivated antioxidant and redox adaptation systems, including the NRF2-KEAP1 axis, glutathione, and thioredoxin pathways (genes downregulated in co-exposure to DPDS and TMZ; Fig. 6). This dependence establishes a state of redox vulnerability, in which the suppression of hyperactivated antioxidant defenses can sensitize cancer cells to oxidative stress-induced death [75, 76].

Thus, strategies that disrupt redox-adaptive pathways, either by inhibiting antioxidant defenses or increasing ROS levels beyond tolerable limits, have emerged as promising therapeutic approaches for glioblastoma [75, 76, 78, 79]. Therefore, we can suggest that co-exposure to DPDS and TMZ induces redox vulnerability in glioblastoma cells by impairing antioxidant defenses, favoring stress-induced cell death.

Inflammation is a well-established hallmark of glioblastoma and plays a central role in the development of an immunosuppressive tumor microenvironment that supports tumor progression and immune evasion [80, 81] In addition, selenoprotein K, whose gene was downregulated in our treatments, is involved in immune cell signaling, and its decreased expression may be associated with impaired immune responsiveness within the tumor microenvironment [69]. In the present study, the suppression of inflammatory mediators (NF-κB, IL-1β, IL-6, TNF-α, and PTGS2) following co-exposure to DPDS and TMZ can be associated with a broader disruption of glioblastoma-associated inflammation and immune evasion [82–84].

In glioblastoma, sustained inflammatory signaling promotes an immunosuppressive tumor microenvironment by supporting tumor-associated macrophage polarization toward a pro-tumoral M2 phenotype and impairing effective antitumor immunity [85–87]. Thus, attenuation of this chronic inflammatory state may contribute to the re-establishment of immune control, limiting inflammation-driven immune evasion rather than suppressing protective immune responses [88–92]. On the other hand, the data found in this study regarding TMZ treatment alone differ from the literature to date, which describes an increase in inflammatory genes such as TNFα, IL6, and NFKB1, in glioblastoma cell lines. This consequence may reflect the different concentrations of TMZ, as well as the different exposure times of treatment performed in each study [93, 94].

Surprisingly, the groups treated with DPDS, alone or in combination with TMZ, showed a reduction in the expression of the evaluated selenoproteins (GPX1, GPX4, TXNRD1 , SELENOS, SELENOK, and DIO2) (Fig. 6B-E and G-K). An increase in selenoproteins was expected because we have some indications that DPDS can be metabolized into inorganic selenium and, consequently, could be incorporated into selenoproteins in the form of the amino acid selenocysteine (SeCys) or produce inorganic Se (particularly HSe- or selenide), which has anticancer activity [16, 19, 95–97]. In fact, in endothelial cells, DPDS increased the expression of GPX4 [96], however, here DPDS caused significant decrease in the mRNA expression of all selenoproteins that were analyzed. The human selenoproteome comprises 25 proteins involved in redox catalysis, playing crucial roles in regulating pathophysiological processes that help prevent cancer progression [21, 98, 99]. TrxR1 is overexpressed in malignant cells and shows a positive correlation with tumor grade in gliomas across critical stages of tumor development [100, 101]. Thus, inhibition of the thioredoxin system has been considered a strategy in the study of antitumor therapies [20]. In our study, we observed that isolated treatment with TMZ reduced TXNRD1 expression, while co-exposure with DPDS potentiated this effect (Fig. 6D). Moreover, SelenoS has been implicated in the progression of GBM [24], and GPX1 overexpression has been correlated with poor prognosis in glioma [21]. Therefore, the decreased expression of these genes in the presence of DPDS and DPDS in combination with TMZ (Fig. 6G and C) suggests that organoselenium compounds may attenuate tumor progression by modulating redox homeostasis and endoplasmic reticulum stress pathways.

The endoplasmic reticulum (ER) regulates protein synthesis, folding, and quality control, known as proteostasis [102–105]. These process can be disrupted under cellular stress conditions, leading to ER stress [106, 107]. Proteostasis is initially maintained through ER-associated degradation (ERAD) [106, 108, 109], while excessive ER stress activates the unfolded protein response (UPR [104, 106, 107, 110]. Concomitantly, ER-resident selenoproteins, including selenoS and selenoK, contribute to ERAD efficiency and UPR modulation, thereby supporting ER homeostasis [16, 111–114]. In this study, the downregulation of SELENOS and SELENOK following DPDS and TMZ treatment (Fig. 6G and I) suggests impaired ERAD capacity, potentially limiting glioblastoma cell adaptation to ER stress and contributing to reduced proliferation or viability. Consistent with the emerging recognition of ERAD and UPR as therapeutic vulnerabilities in cancer [104, 115–117], these findings highlight ER-resident selenoproteins as promising pharmacological targets.

In addition, glioblastoma cells maintain prolonged UPR activation, which has been associated with the regulation of epithelial–mesenchymal transition (EMT), thereby contributing to increased invasiveness and tumor progression [104, 106, 118–121]. In this context, we highlight that co-treatment with DPDS and TMZ reduced the expression of SNAIL1 (Fig. 9E), a key transcription factor involved in the control of EMT and a major driver of cellular invasion and migration [122, 123].

Taken together, we hypothesize that the combination of DPDS and TMZ may have negative effects in U87-MG glioblastoma cells by modulating oxidative stress and inflammatory profile; specifically, DPDS and TMZ downregulated the mRNA expression of pro-inflammatory proteins and oxidative stress markers (IL6, IL1B, NFKB1, PTGS2, TNFA, NFKB1, GPX1, GPX4, SELENOS, SELENOK, SELENOI, KEAP1, and TXNRD1). To verify the scope of the downregulated profile of the combination of DPDS and TMZ, we evaluated the expression of proteins related to apoptosis, hypoxia response, epigenetic regulators, cell cycle, DNA damage response, and epithelial–mesenchymal transition (EMT).

Our findings demonstrated that co-exposure to DPDS and TMZ led to a downregulation of genes related to apoptosis (BAX, BAD, and BCL-2), hypoxia response (HIF1A), and epigenetic regulation (HDAC4), which were broadly reduced (Figs. 8 and 9), suggesting interference with cell survival mechanisms [26, 124–126].

NEK1 and NEK2 are members of the NIMA-related kinase (NEK) family, which regulates key cellular processes including cell cycle control and DNA damage response, and whose members are broadly implicated in cancer biology [127–131]. NEK1 is upregulated in human glioma tissues and cell lines, with higher expression correlating with tumor grade and poor patient survival, while NEK2 is frequently overexpressed in glioblastoma and other cancers, where it serves as a diagnostic and prognostic marker [130, 132].

In relation to cancer cell cycle regulation and epithelial–mesenchymal transition (EMT), the suppression of NEK1, NEK2, and SNAIL1 observed in our study may indicate critical alterations in key mechanisms underlying cancer aggressiveness [129]. These kinases and transcription factors are not merely markers of proliferation and migration but active regulators of genomic stability, mitotic fidelity, and invasive behavior in glioma cells [130, 131, 133]. Thus, their coordinated downregulation following DPDS and TMZ co-exposure (Fig. 9) is consistent with impaired cell cycle progression, reduced migratory capacity, and attenuation of invasion-associated programs [130, 131, 133].

The increasing resistance of tumors to conventional therapies has driven the search for novel anticancer agents. García-López et al., [106], Kar, [134], Mai, [135], Oliveira, [136]. Organoselenium compounds have emerged as promising candidates due to their ability to modulate redox balance, cell cycle progression, and cell death pathways [26, 32, 66, 137–139]. However, their biological effects are highly dose and context dependent, often reflecting a narrow therapeutic window [13].

Although DPDS has been extensively studied for its antioxidant and cytoprotective properties [17, 33, 96, 140], evidence of its anticancer activity, particularly in glioblastoma, remains limited [45]. In this context, the present study provides relevant information on the effects of DPDS on glioblastoma cells, while reinforcing the need for more in vitro and in vivo studies to define its mode of action and safety profile before translational application.

Finally, the present study represents an initial investigation into the potential of DPDS as an anti-glioblastoma agent and as an adjuvant to TMZ therapy. Although we performed a broad gene expression analysis covering key pathways involved in tumor survival and progression, a limitation of this work is that the analyses were restricted to mRNA levels, which may not directly reflect protein abundance or functional activity [141]. Post-transcriptional and post-translational regulatory mechanisms can lead to discrepancies between mRNA and protein expression, underscoring the need for future studies incorporating protein-level and functional analyses [142].

## Conclusion

This study demonstrates, for the first time in the literature, the effects of the diphenyl diselenide (DPDS) in combination with temozolomide (TMZ) on human glioblastoma U87-MG cells. Co-exposure significantly reduced cell viability, density, and migration. Furthermore, it promoted a general suppression of multiple pathways critical for glioblastoma cells’ survival, including the antioxidant response, oxidative stress, inflammation, apoptosis, epigenetic regulation, hypoxia response, cell cycle regulation, and epithelial-mesenchymal transition (EMT). Therefore, the results highlight the potential of DPDS as an adjuvant in TMZ-based chemotherapy; however, further investigation is needed to determine the precise mode of action of the combination of these two molecules. Furthermore, this study provides new evidence for future studies seeking to evaluate the potential of selenium-containing compounds in cancer research.

## Data Availability

All tables containing data generated from our research, i.e., raw results for analysis, will be provided upon request to the corresponding author.

## Abbreviations

DPDS: Diphenyl Diselenide
TMZ: Temozolomide
ROS: Reactive Oxygen Species
CAT: Catalase
SOD: Superoxide dismutase
NRF2 (NFE2L2): Nuclear factor erythroid 2–related factor 2
KEAP1: Kelch–like ECH–associated protein 1
TXNRD1: Thioredoxin Reductase 1
TXNRD2: Thioredoxin Reductase 2
GPX1: Glutathione peroxidase 1
GPX4: Glutathione peroxidase 4
SELENOS: Selenoprotein S
SELENOP: Selenoprotein P
SELENOI: Selenoprotein I
SELENOK: Selenoprotein K
DIO2: Deiodinase, iodothyronine type II
NFKB1: Nuclear factor kappa B subunit 1
IL6: Interleukin 6
IL1B (IL: 1β)–Interleukin 1 beta
TNFα (TNF): Tumor necrosis factor alpha
PTGS2 (COX: 2)–Prostaglandin–endoperoxide synthase 2
BAX: Bcl–2–associated X protein
BAD: Bcl–2–associated death promoter
BCL2: B–cell lymphoma 2
NEK1: NIMA related kinase 1
NEK2: NIMA related kinase 2
SNAI1: Snail family transcriptional repressor 1
HDAC4: Histone deacetylase 4
HIF1A: Hypoxia–inducible factor 1 alpha
ACTB: Beta–actin

## References

[1] Cui X, Wang Y, Zhou J, Wang Q, Kang C (2023) Expert opinion on translational research for advanced glioblastoma treatment. Cancer Biol Med 20:344–352. 10.20892/j.issn.2095-3941.2023.0012 37092846 10.20892/j.issn.2095-3941.2023.0012 PMC10246444

[2] Tyagi A, Wu SY, Watabe K (2022) Metabolism in the progression and metastasis of brain tumors. Cancer Lett. 10.1016/j.canlet.2022.215713 35513201 10.1016/j.canlet.2022.215713 PMC9999298

[3] Weller M, Wick W, Aldape K, Brada M, Berger M, Pfister SM, Nishikawa R, Rosenthal M, Wen PY, Stupp R, Reifenberger G (2015) Glioma. Nat Rev Dis Primers. 10.1038/nrdp.2015.17 27188790 10.1038/nrdp.2015.17

[4] Wesseling P, Kros JM, Jeuken JWM (2011) The pathological diagnosis of diffuse gliomas: towards a smart synthesis of microscopic and molecular information in a multidisciplinary context. Diagn Histopathol 17:486–494. 10.1016/j.mpdhp.2011.08.005

[5] Sipos D, Raposa BL, Freihat O, Mekis N, Cornacchione P (2025) Glioblastoma : clinical presentation, multidisciplinary management, and long-term outcomes. Cancers (Basel). 10.3390/cancers17010146 39796773 10.3390/cancers17010146 PMC11719842

[6] Tsuji S, Nakamura S, Maoka T, Yamada T, Imai T, Ohba T, Yako T, Hayashi M, Endo K, Saio M, Hara H, Shimazawa M (2020) Antitumour effects of astaxanthin and adonixanthin on glioblastoma. Mar Drugs 18:1–16. 10.3390/md18090474 10.3390/md18090474 PMC7551886 32962073

[7] Avci NG, Ebrahimzadeh-Pustchi S, Akay YM, Esquenazi Y, Tandon N, Zhu JJ, Akay M (2020) NF-κB inhibitor with Temozolomide results in significant apoptosis in glioblastoma via the NF-κB(p65) and actin cytoskeleton regulatory pathways. Sci Rep 10:1–14. 10.1038/s41598-020-70392-5 32770097 10.1038/s41598-020-70392-5 PMC7414229

[8] Jezierzański M, Nafalska N, Stopyra M, Furgoł T, Miciak M, Kabut J, Gisterek-Grocholska I (2024) Temozolomide (TMZ) in the treatment of glioblastoma multiforme—a literature review and clinical outcomes. Curr Oncol 31:3994–4002. 10.3390/curroncol31070296 39057168 10.3390/curroncol31070296 PMC11275351

[9] Herbener VJ, Burster T, Goreth A, Pruss M, von Bandemer H, Baisch T, Fitzel R, Siegelin MD, Karpel-Massler G, Debatin KM, Westhoff MA, Strobel H (2020) Considering the experimental use of Temozolomide in glioblastoma research. Biomedicines 8:1–29. 10.3390/BIOMEDICINES8060151 10.3390/biomedicines8060151 PMC7344626 32512726

[10] Thanh HD, Lee S, Nguyen TT, Huu TN, Ahn EJ, Cho SH, Kim MS, Moon KS, Jung C (2024) Temozolomide promotes matrix metalloproteinase 9 expression through p38 MAPK and JNK pathways in glioblastoma cells. Sci Rep 14:1–13. 10.1038/s41598-024-65398-2 38906916 10.1038/s41598-024-65398-2 PMC11192740

[11] Li H, Wu Y, Chen Y, Lv J, Qu C, Mei T, Zheng Y, Ye C, Li F, Ge S, Yao A, Jia L (2025) Overcoming temozolomide resistance in glioma: recent advances and mechanistic insights. Acta Neuropathol Commun. 10.1186/s40478-025-02046-4 40468460 10.1186/s40478-025-02046-4 PMC12139195

[12] Teraiya M, Perreault H, Chen VC (2023) An overview of glioblastoma multiforme and temozolomide resistance: can LC-MS-based proteomics reveal the fundamental mechanism of temozolomide resistance? Front Oncol. 10.3389/fonc.2023.1166207 37182181 10.3389/fonc.2023.1166207 PMC10169742

[13] Radomska D, Czarnomysy R, Radomski D, Bielawski K (2021) Selenium compounds as novel potential anticancer agents. Int J Mol Sci 22:1–27. 10.3390/ijms22031009 10.3390/ijms22031009 PMC7864035 33498364

[14] Hatfield DL, Tsuji PA, Carlson BA, Gladyshev VN (2014) Selenium and selenocysteine: roles in cancer, health and development. Trends Biochem Sci 39:112–120. 10.1016/j.tibs.2013.12.007 24485058 10.1016/j.tibs.2013.12.007 PMC3943681

[15] Minich WB (2022) Selenium metabolism and biosynthesis of selenoproteins in the human body. Biochem 87:S168–S177. 10.1134/S0006297922140139 35501994 10.1134/S0006297922140139 PMC8802287

[16] Shahidin, Wang Y, Wu Y, Chen T, Wu X, Yuan W, Zhu Q, Wang X, Zi C (2025) Selenium and Selenoproteins: Mechanisms, Health Functions, and Emerging Applications. Molecules 30:1–35. ttps://doi.org/10.3390/molecules30030437 10.3390/molecules30030437 PMC11820089 39942544

[17] Barbosa N, Nogueira CW, Guecheva TN, De Lourdes Bellinaso M, Rocha JBT (2008) Diphenyl diselenide supplementation delays the development of N-nitroso-N-methylurea-induced mammary tumors. Arch Toxicol 82:655–663. 10.1007/s00204-007-0271-9 18074119 10.1007/s00204-007-0271-9

[18] Barchielli G, Capperucci A, Tanini D (2022) The role of selenium in pathologies: an updated review. Antioxidants. 10.3390/antiox11020251 35204134 10.3390/antiox11020251 PMC8868242

[19] Hariharan S, Dharmaraj S (2020) Selenium and selenoproteins: it’s role in regulation of inflammation. Inflammopharmacology 28:667–695. ttps://doi.org/10.1007/s10787-020-00690-x 32144521 10.1007/s10787-020-00690-x PMC7222958

[20] Bramatti I, Aschner M, Branco V, Carvalho C (2024) Exposure of human glioblastoma cells to thimerosal inhibits the thioredoxin system and decreases tumor growth-related factors. Toxicol Appl Pharmacol 484:116844. 10.1016/j.taap.2024.116844 38325586 10.1016/j.taap.2024.116844

[21] Chen X, Fu G, Li L, Zhao Q, Ke Z, Zhang R (2022) Selenoprotein GPX1 is a prognostic and chemotherapy-related biomarker for brain lower grade glioma. J Trace Elem Med Biol 74:127082. 10.1016/j.jtemb.2022.127082 36155420 10.1016/j.jtemb.2022.127082

[22] Madabeni A, Dalla Tiezza M, Omage FB, Nogara PA, Bortoli M, Rocha JBT, Orian L (2020) Chalcogen–mercury bond formation and disruption in model Rabenstein’s reactions: a computational analysis. J Comput Chem 41:2045–2054. 10.1002/jcc.26371 32656797 10.1002/jcc.26371

[23] Pires V, Bramatti I, Aschner M, Branco V, Carvalho C (2022) Thioredoxin reductase inhibitors as potential antitumors: mercury compounds efficacy in glioma cells. Front. Mol. Biosci. 9:1–11. 10.3389/fmolb.2022.889971 10.3389/fmolb.2022.889971 PMC9260667 35813817

[24] Wang Y, Qu K, Xia Z, Qi M, Du X, Ke Z, Zhang R (2024) Selenoprotein S (SELENOS) is a potential prognostic biomarker for brain lower grade glioma. J Trace Elem Med Biol 86:127539. 10.1016/j.jtemb.2024.127539 39378668 10.1016/j.jtemb.2024.127539

[25] Wu W, Li D, Feng X, Zhao F, Li C, Zheng S, Lyu J (2021) A pan-cancer study of selenoprotein genes as promising targets for cancer therapy. BMC Med Genomics 14:1–14. 10.1186/s12920-021-00930-1 33706760 10.1186/s12920-021-00930-1 PMC7948377

[26] Berthier S, Arnaud J, Champelovier P, Col E, Garrel C, Cottet C, Boutonnat J, Laporte F, Faure P, Hazane-Puch F (2017) Anticancer properties of sodium selenite in human glioblastoma cell cluster spheroids. J Trace Elem Med Biol 44:161–176. 10.1016/j.jtemb.2017.04.012 28965572 10.1016/j.jtemb.2017.04.012

[27] Harmanci D, Erbayraktar Z, Sayin O, Guner GA (2017) In vitro effects of selenium on human glioblastoma multiforme cell lines: a preliminary study. Acta Clin Croat 56:48–57. 10.20471/acc.2017.56.01.08 29120131 10.20471/acc.2017.56.01.08

[28] Zhong M, Bi Y, Zhang J, Li S, Nie Y, He X (2025) Synthesis, anticancer activity and molecular modeling of new NSAIDs-Ebselen derivatives as potential TrxR1 inhibitor. Results Chem 18:102802. 10.1016/j.rechem.2025.102802

[29] Cheng Y, Sk UH, Zhang Y, Ren X, Zhang L, Huber-Keener KJ, Sun YW, Liao J, Amin S, Sharma AK, Yang JM (2012) Rational incorporation of selenium into temozolomide elicits superior antitumor activity associated with both apoptotic and autophagic cell death. PLoS One 7:1–10. 10.1371/journal.pone.0035104 10.1371/journal.pone.0035104 PMC3320619 22496897

[30] Baldissera MD, Souza CF, da Silva AS, Henn AS, Flores EMM, Baldisserotto B (2020) Diphenyl diselenide dietary supplementation alleviates behavior impairment and brain damage in grass carp (*Ctenopharyngodon idella*) exposed to methylmercury chloride. Comp. Biochem. Physiol. Part - C Toxicol. Pharmacol. 229:108674. 10.1016/j.cbpc.2019.108674 10.1016/j.cbpc.2019.108674 31760078

[31] de Freitas AS, Funck VR, Rotta M, Bohrer D, Mörschbächer V, Puntel RL, Nogueira CW, Farina M, Aschner M, Rocha JBT (2009) Diphenyl diselenide, a simple organoselenium compound, decreases methylmercury-induced cerebral, hepatic and renal oxidative stress and mercury deposition in adult mice. Brain Res Bull 79:77–84. 10.1016/j.brainresbull.2008.11.001 19047014 10.1016/j.brainresbull.2008.11.001

[32] Nogueira CW, Rocha JB (2010) Diphenyl diselenide a janus-faced molecule. J Braz Chem Soc 21:2055–2071

[33] Quispe RL, Jaramillo ML, Galant LS, Engel D, Dafre AL, Teixeira da Rocha JB, Radi R, Farina M, de Bem AF (2019) Diphenyl diselenide protects neuronal cells against oxidative stress and mitochondrial dysfunction: involvement of the glutathione-dependent antioxidant system. Redox Biol 20:118–129. 10.1016/j.redox.2018.09.014 30308475 10.1016/j.redox.2018.09.014 PMC6176650

[34] Rieder GS, Braga MM, Hur B, Emerson MM, Gabriela SS, André E, Diogo C, Jeferson LO, Diogo LF, João OGS, Rocha BT (2024) Diphenyl diselenide attenuates mitochondrial damage during initial hypoxia and enhances resistance to recurrent hypoxia. Neurotox Res. 10.1007/s12640-024-00691-6 38332435 10.1007/s12640-024-00691-6

[35] Wildner G, Tucci AR, Prestes ADS, Muller T, Rosa S, Borba NRR, Ferreira VN, Rocha T, Miranda MD, Barbosa NV (2023) Ebselen and diphenyl diselenide inhibit SARS-CoV-2 replication at non-toxic concentrations to. Hum Cell Lines 2:1–16 10.3390/vaccines11071222 PMC10384302 37515038

[36] Brüning CA, Prigol M, Luchese C, Jesse CR, Duarte MMMF, Roman SS, Nogueira CW (2012) Protective effect of diphenyl diselenide on ischemia and reperfusion-induced cerebral injury: involvement of oxidative stress and pro-inflammatory cytokines. Neurochem Res 37:2249–2258. 10.1007/s11064-012-0853-7 22846969 10.1007/s11064-012-0853-7

[37] Fiuza B, Subelzú N, Calcerrada P, Straliotto MR, Piacenza L, Cassina A, Rocha JBT, Radi R, De Bem AF, Peluffo G (2015) Impact of SIN-1-derived peroxynitrite flux on endothelial cell redox homeostasis and bioenergetics: protective role of diphenyl diselenide via induction of peroxiredoxins. Free Radic Res 49:122–132. 10.3109/10715762.2014.983096 25373783 10.3109/10715762.2014.983096

[38] Wang X, Li C, Huan Y, Cao H, Sun S, Lei L, Liu Q, Liu S, Ji W, Huang K, Shen Z, Zhou J (2021) Diphenyl diselenide ameliorates diabetic nephropathy in streptozotocin-induced diabetic rats via suppressing oxidative stress and inflammation. Chem Biol Interact. 10.1016/j.cbi.2021.109427 33639173 10.1016/j.cbi.2021.109427

[39] Liu Q, Li G, Li R, Shen J, He Q, Deng L, Zhang C, Zhang J (2010) IL-6 promotion of glioblastoma cell invasion and angiogenesis in U251 and T98G cell lines. J Neurooncol 100:165–176. 10.1007/s11060-010-0158-0 20361349 10.1007/s11060-010-0158-0

[40] Mahalingaiah PKS, Singh KP (2014) Chronic oxidative stress increases growth and tumorigenic potential of MCF-7 breast cancer cells. PLoS One. 10.1371/journal.pone.0087371 24489904 10.1371/journal.pone.0087371 PMC3905021

[41] Liang X, Wang Z, Dai Z, Liu J, Zhang H, Wen J, Zhang N, Zhang J, Luo P, Liu Z, Liu Z, Cheng Q (2024) Oxidative stress is involved in immunosuppression and macrophage regulation in glioblastoma. Clin Immunol 258:109802. 10.1016/j.clim.2023.109802 37866784 10.1016/j.clim.2023.109802

[42] Tafani M, Di Vito M, Frati A, Pellegrini L, De Santis E, Sette G, Eramo A, Sale P, Mari E, Santoro A, Raco A, Salvati M, De Maria R, Russo MA (2011) Pro-inflammatory gene expression in solid glioblastoma microenvironment and in hypoxic stem cells from human glioblastoma. J Neuroinflammation 8:1–16. 10.1186/1742-2094-8-32 21489226 10.1186/1742-2094-8-32 PMC3098164

[43] Adedara IA, Abolaji AO, Rocha JBT, Farombi EO (2016) Diphenyl diselenide protects against mortality, locomotor deficits and oxidative stress in *Drosophila melanogaster* model of manganese-induced neurotoxicity. Neurochem Res 41:1430–1438 26875733 10.1007/s11064-016-1852-x

[44] Rieder GS, Duarte T, Delgado CP, Rodighiero A, Nogara PA, Orian L, Aschner M, Corte CLD, Rocha JD (2024) Interplay between diphenyl diselenide and copper: impact on *D. melanogaster* survival, behavior, and biochemical parameters. Comp Biochem Physiol C Toxicol Pharmacol 109403. 10.1016/j.cbpc.2024.109899 10.1016/j.cbpc.2024.109899 38518983

[45] Ferreira LM, Azambuja JH, da Silveira EF, Marcondes Sari MH, da Cruz Weber Fulco B, Costa Prado V, Gelsleichter NE, Beckenkamp LR, da Cruz Fernandes M, Spanevello RM, Wink MR, de Cassia Sant Anna Alves R, Nogueira CW, Braganhol E, Cruz L (2019) Antitumor action of diphenyl diselenide nanocapsules: in vitro assessments and preclinical evidence in an animal model of glioblastoma multiforme. J Trace Elem Med Biol 55:180–189. 10.1016/j.jtemb.2019.06.010 31345356 10.1016/j.jtemb.2019.06.010

[46] Mosmann T (1983) Rapid colorimetric assay for cellular growth and survival: application to proliferation and cytotoxicity assays. J Immunol Methods 65:55–63. 10.1016/0022-1759(83)90303-4 6606682 10.1016/0022-1759(83)90303-4

[47] Cwiklowska K, Westhoff MA, Freisinger S, Dwucet A, Halatsch ME, Knippschild U, Debatin KM, Schirmbeck R, Winiarski L, Oleksyszyn J, Wirtz CR, Burster T (2018) Viability of glioblastoma stem cells is effectively reduced by diisothiocyanate-derived mercapturic acids. Oncol Lett 16:6181–6187. 10.3892/ol.2018.9347 30344758 10.3892/ol.2018.9347 PMC6176379

[48] Blois J, Smith A, Josephson L (2011) The slow cell death response when screening chemotherapeutic agents. Cancer Chemother Pharmacol 68:795–803. ttps://doi.org/10.1007/s00280-010-1549-9.The 21193989 10.1007/s00280-010-1549-9 PMC3210817

[49] Okur ME, Karakaş N, Karadağ AE, Yilmaz R, Demirci F (2019) In vitro cytotoxicity evaluation of *Marrubium vulgare* L. methanol extract. J Res Pharm 23:711–718. 10.12991/jrp.2019.180

[50] Pekmez M, Kılcı C (2022) The effect of temozolomide on Hsp60 and Hsp70 expression in extracellular vesicles derived from U87MG glioma cells. Turk J Biochem 47:85–95. 10.1515/tjb-2021-0111

[51] Poon MTC, Bruce M, Simpson JE, Hannan CJ, Brennan PM (2021) Temozolomide sensitivity of malignant glioma cell lines – a systematic review assessing consistencies between in vitro studies. BMC Cancer 21:1–9. 10.1186/s12885-021-08972-5 34794398 10.1186/s12885-021-08972-5 PMC8600737

[52] Lowry OH, Rosebrough NJ, Farr AL, Randall RJ (1951) Protein measurement with the Folin phenol reagent. J Biol Chem 193:265–275. 10.1016/0304-3894(92)87011-4 14907713

[53] LeBel CP, Ischiropoulos H, Bondy SC (1992) Evaluation of the probe 2’,7’-dichlorofluorescin as an indicator of reactive oxygen species formation and oxidative stress. Chem Res Toxicol 5:227–231. 10.1021/tx00026a012 1322737 10.1021/tx00026a012

[54] Aebi H (1984) [13] catalase in vitro. Methods Enzymol 105:121–126. 10.1016/S0076-6879(84)05016-3 6727660 10.1016/s0076-6879(84)05016-3

[55] Marklund SL (1985) Superoxide dismutase isoenzymes in tissues and plasma from New Zealand black mice, nude mice and normal BALB/c mice. Mutat Res-Fundam Mol Mech Mutagen 148:129–134. 10.1016/0027-5107(85)90216-7 10.1016/0027-5107(85)90216-7 3969077

[56] Bellaver B, Bobermin LD, Souza DG, Rodrigues MDN, de Assis AM, Wajner M, Gonçalves CA, Souza DO, Quincozes-Santos A (2016) Signaling mechanisms underlying the glioprotective effects of resveratrol against mitochondrial dysfunction. Biochim. Biophys. Acta - Mol. Basis Dis. 1862:1827–1838. 10.1016/j.bbadis.2016.06.018 10.1016/j.bbadis.2016.06.018 27373419

[57] Livak KJ, Schmittgen TD (2001) Analysis of relative gene expression data using real-time quantitative PCR and the 2-ΔΔCT method. Methods 25:402–408. 10.1006/meth.2001.1262 11846609 10.1006/meth.2001.1262

[58] Kricha A, Bouchmaa N, Ben Mkaddem S, Abbaoui A, Ben Mrid R, El Fatimy R (2024) Glioblastoma-associated macrophages: a key target in overcoming glioblastoma therapeutic resistance. Cytokine Growth Factor Rev 80:97–108. 10.1016/j.cytogfr.2024.10.009 39510901 10.1016/j.cytogfr.2024.10.009

[59] Pouyan A, Ghorbanlo M, Eslami M, Jahanshahi M, Ziaei E, Salami A, Mokhtari K, Shahpasand K, Farahani N, Meybodi TE, Entezari M, Taheriazam A, Hushmandi K, Hashemi M (2025) Glioblastoma multiforme: insights into pathogenesis, key signaling pathways, and therapeutic strategies. Mol Cancer. 10.1186/s12943-025-02267-0 40011944 10.1186/s12943-025-02267-0 PMC11863469

[60] Hotchkiss KM, Sampson JH (2021) Temozolomide treatment outcomes and immunotherapy efficacy in brain tumor. J Neurooncol 151:55–62. 10.1007/s11060-020-03598-2 32813186 10.1007/s11060-020-03598-2 PMC9833842

[61] Mohammed S, Dinesan M, Ajayakumar T (2022) Survival and quality of life analysis in glioblastoma multiforme with adjuvant chemoradiotherapy: a retrospective study. Rep Pract Oncol Radiother 27:1026–1036. 10.5603/RPOR.a2022.0113 36632307 10.5603/RPOR.a2022.0113 PMC9826661

[62] Zarei Shandiz S, Erfani B, Hashemy SI (2024) Protective effects of silymarin in glioblastoma cancer cells through redox system regulation. Mol Biol Rep 51:1–7. 10.1007/s11033-024-09658-4 10.1007/s11033-024-09658-4 38833199

[63] Diez BD, Statkevich P, Zhu Y, Abutarif MA, Xuan F, Kantesaria B, Cutler D, Cantillon M, Schwarz M, Pallotta MG, Ottaviano FH (2010) Evaluation of the exposure equivalence of oral versus intravenous temozolomide. Cancer Chemother Pharmacol 65:727–734. 10.1007/s00280-009-1078-6 19641919 10.1007/s00280-009-1078-6 PMC2808524

[64] Tanaka F, Irie K, Fukui N, Horii R, Imamura H, Hirabatake M, Ikesue H, Muroi N, Fukushima S, Sakai N, Hashida T (2023) Pharmacokinetics of Temozolomide in a Patient with Glioblastoma Undergoing Hemodialysis: A Short Communication. Ther Drug Monit 45:823–826. 10.1097/FTD.0000000000001125 37646650 10.1097/FTD.0000000000001125 PMC10635330

[65] da Costa NS, Lima LS, Oliveira FAM, Galiciolli MEA, Manzano MI, Garlet QI, Irioda AC, Oliveira CS (2023) Antiproliferative Effect of Inorganic and Organic Selenium Compounds in Breast Cell Lines. Biomedicines 11:1–13. 10.3390/biomedicines11051346 10.3390/biomedicines11051346 PMC10216490 37239017

[66] Díaz M, González R, Plano D, Palop JA, Sanmartín C, Encío I (2018) A diphenyldiselenide derivative induces autophagy via JNK in HTB-54 lung cancer cells. J Cell Mol Med 22:289–301. 10.1111/jcmm.13318 28922542 10.1111/jcmm.13318 PMC5742718

[67] Hammouda MM, Shaaban S, Ba-Ghazal H, Dawood AF, Zeidan MA, Al Khatib AO, Alatawi FS, Alomari KB, Sharaky M, Alaasar M, Al-Karmalawy AA (2025) Novel diselenide bis-Schiff bases tagged with diphenyl ethers: promising candidates for selective treatment of hypopharyngeal and breast adenocarcinoma. Bioorg Chem 163:108670. 10.1016/j.bioorg.2025.108670 40554888 10.1016/j.bioorg.2025.108670

[68] Zhou NJ, Bao WQ, Zhang CF, Jiang ML, Liang TL, Ma GY, Liu L, Pan HD, Li RZ (2025) Immunometabolism and oxidative stress: roles and therapeutic strategies in cancer and aging. NPJ Aging. 10.1038/s41514-025-00250-z 40593697 10.1038/s41514-025-00250-z PMC12217376

[69] Marciel MP, Hoffmann PR (2019) Molecular mechanisms by which selenoprotein K regulates immunity and cancer. Biol Trace Elem Res 192:60–68. 10.1007/s12011-019-01774-8 31187393 10.1007/s12011-019-01774-8 PMC6801056

[70] Marciel MP, Khadka VS, Deng Y, Kilicaslan P, Pham A, Bertino P, Lee K, Chen S, Glibetic N, Hoffmann FKW, Matter ML, Hoffmann PR (2018) Selenoprotein k deficiency inhibits melanoma by reducing calcium flux required for tumor growth and metastasis. Oncotarget 9:13407–13422. 10.18632/oncotarget.24388 29568366 10.18632/oncotarget.24388 PMC5862587

[71] Kim EH, Sohn S, Kwon HJ, Kim SU, Kim MJ, Lee SJ, Choi k (2007) Sodium selenite induces superoxide-mediated mitochondrial damage and subsequent autophagic cell death in malignant glioma cells. Cancer Res 67:6314–6324. 10.1158/0008-5472.CAN-06-4217 17616690 10.1158/0008-5472.CAN-06-4217

[72] Wang X, Huan Y, Liu S, Li C, Cao H, Lei L, Liu Q, Ji W, Sun S, Huang K, Zhou J, Shen Z (2022) Diphenyl diselenide alleviates tert-butyl hydrogen peroxide-induced oxidative stress and lipopolysaccharide-induced inflammation in rat glomerular mesangial cells. Int J Mol Sci. 10.3390/ijms231911215 36232514 10.3390/ijms231911215 PMC9570341

[73] Mancini G, Raniel Straliotto M, da Rocha JBT, de Bem AF (2014) Diphenyl diselenide improves the antioxidant response via activation of the Nrf-2 pathway in macrophage cells. Free Radic Biol Med 75:40. 10.1016/j.freeradbiomed.2014.10.788 26461369 10.1016/j.freeradbiomed.2014.10.788

[74] Liu S, Liu J (2025) Oxidative Stress : Signaling Pathways, Biological Functions, and Disease. MedComm Rev 6:e70268. 10.1002/mco2.70268 10.1002/mco2.70268 PMC12209598 40599237

[75] Ranbhise JS, Singh MK, Ju S, Han S, Yun HR, Kim SS, Kang I (2025) The Redox Paradox : Cancer ’ s Double-Edged Sword for Malignancy and Therapy. Antioxidants 14:1–27. 10.3390/antiox14101187 10.3390/antiox14101187 PMC12561025 41154495

[76] Kar E, Övenler Z, Hacıoğlu C, Kar F (2025) Boric acid induces oxidative damage and apoptosis through SEMA3A / PLXNA1 / NRP1 signalling pathway in U251 glioblastoma cell. J Cell Mol Med. 10.1111/jcmm.70578 40318008 10.1111/jcmm.70578 PMC12049150

[77] Olufunmilayo EO, Gerke-Duncan MB, Holsinger RMD (2023) Oxidative stress and antioxidants in neurodegenerative disorders. Antioxidants 12:1–30. 10.3390/antiox12020517 10.3390/antiox12020517 PMC9952099 36830075

[78] Hacioglu C, Kar F (2023) Capsaicin induces redox imbalance and ferroptosis through ACSL4/GPx4 signaling pathways in U87-MG and U251 glioblastoma cells. Metab Brain Dis 38:393–408. ttps://doi.org/10.1007/s11011-022-00983-w 35438378 10.1007/s11011-022-00983-w

[79] Tuncer C, Hacioglu C (2025) Notch1 and Major Vault Proteins Modulate Temozolomide Resistance in Glioblastoma. J Cell Mol Med 29:e70474. 10.1111/jcmm.70474 40100070 10.1111/jcmm.70474 PMC11916442

[80] Greten FR, Grivennikov SI (2019) Inflammation and Cancer : Triggers, Mechanisms, and Consequences. Immunity 51:27–41. 10.1016/j.immuni.2019.06.025 31315034 10.1016/j.immuni.2019.06.025 PMC6831096

[81] Medikonda R, Abikenari M, Schonfeld E, Lim M (2025) The metabolic orchestration of immune evasion in glioblastoma: from molecular perspectives to therapeutic vulnerabilities. Cancers (Basel). 17:1881. 10.3390/cancers17111881 40507361 10.3390/cancers17111881 PMC12153820

[82] Basheer AS, Abas F, Othman I, Naidu R (2021) Role of inflammatory mediators, macrophages, and neutrophils in glioma maintenance and progression: mechanistic understanding and potential therapeutic applications. Cancers. 10.3390/cancers13164226 34439380 10.3390/cancers13164226 PMC8393628

[83] Jarmuzek P, Defort P, Kot M, Wawrzyniak-gramacka E, Morawin B, Zembron-lacny A (2023) Cytokine profile in development of glioblastoma in relation to healthy individuals. Int J Mol Sci 24:16206. 10.3390/ijms242216206 38003396 10.3390/ijms242216206 PMC10671437

[84] Yeung YT, Mcdonald KL, Grewal T, Munoz L (2013) Interleukins in glioblastoma pathophysiology : implications for therapy. Br J Pharmacol 168:591–606. 10.1111/bph.12008 23062197 10.1111/bph.12008 PMC3579281

[85] Basak U, Sarkar T, Mukherjee S, Chakraborty S, Dutta A, Dutta S, Nayak D, Kaushik S, Das T, Sa G (2023) Tumor-associated macrophages : an effective player of the tumor microenvironment. Front Immunol. 10.3389/fimmu.2023.1295257 38035101 10.3389/fimmu.2023.1295257 PMC10687432

[86] Sadeghi M, Dehnavi S, Sharifat M, Amiri AM, Khodadadi A (2024) Innate immune cells : Key players of orchestra in modulating tumor microenvironment ( TME ). Heliyon 10:e27480. 10.1016/j.heliyon.2024.e27480 38463798 10.1016/j.heliyon.2024.e27480 PMC10923864

[87] Zhao W, Zhang Z, Xie M, Ding F, Zheng X, Sun S, Du J (2025) Exploring tumor-associated macrophages in glioblastoma: from diversity to therapy. NPJ Precis Oncol 9:1–18. 10.1038/s41698-025-00920-x 40316746 10.1038/s41698-025-00920-x PMC12048723

[88] Hacioglu C (2024) Capsaicin enhances temozolomide-resistant glioblastoma cells’ chemosensitivity and ferroptosis through FHOD1/IRF2 downregulation. J Food Biochem 2024:1–11. 10.1155/2024/8464817

[89] Mukherjee S, Fried A, Hussaini R, White R, Baidoo J, Yalamanchi S, Banerjee P (2018) Phytosomal curcumin causes natural killer cell-dependent repolarization of glioblastoma (GBM) tumor-associated microglia / macrophages and elimination of GBM and GBM stem cells. J Exp Clin Cancer Res 37:1–18. 10.1186/s13046-018-0792-5 30041669 10.1186/s13046-018-0792-5 PMC6058381

[90] Nasir M, Malik H, Ali S, Ali A, Alanzi AR, Atif M, Alharbi HA, Wang B, Raza M, Maqbool T, Anjum I, Jahan S, Alshammari S, Solre G (2025) Citronellol induces apoptosis via differential regulation of caspase- ­ κB, and JAK2 signaling pathways in glioblastoma cell line. Food Sci Nutr 13:1–18. 10.1002/fsn3.4678 10.1002/fsn3.4678 PMC11717069 39803280

[91] Ramar V, Guo S, Wang G, Liu M (2025) The pivotal role of NF- κ b in glioblastoma : mechanisms of activation and therapeutic implications. Int. J. Sci. 26:1–24. 10.3390/ijms26167883 10.3390/ijms26167883 PMC12387013 40869207

[92] Yang F, He Z, Duan H, Zhang D, Li J, Yang H, Dorsey JF, Zou W, Nabavizadeh SA, Bagley SJ, Abdullah K, Brem S, Zhang L, Xu X, Byrne KT, Vonderheide RH, Gong Y, Fan Y (2021) Synergistic immunotherapy of glioblastoma by dual targeting of IL-6 and CD40. Nat Commun. 10.1038/s41467-021-23832-3 10.1038/s41467-021-23832-3 PMC8187342 34103524

[93] Kapteijn MY, Zwaan S, ter Linden E, Laghmani EH, van den Akker RFP, Rondon AMR, van der Zanden SY, Neefjes J, Versteeg HH, Buijs JT (2023) Temozolomide and lomustine induce tissue factor expression and procoagulant activity in glioblastoma cells *in vitro*. Cancers (Basel). 15:1–13. 10.3390/cancers15082347 10.3390/cancers15082347 PMC10137012 37190275

[94] Liu F, Zhou Q, Jiang H, Zhang T, Miao C, Xu X, Wu J, Yin S, Xu S, Peng J, Gao P, Cao X, Pan F, He X, Chen XQ (2023) Piperlongumine conquers temozolomide chemoradiotherapy resistance to achieve immune cure in refractory glioblastoma via boosting oxidative stress-inflamation-CD8+-T cell immunity. J. Exp. Clin. Cancer Res 42:1–19. 10.1186/s13046-023-02686-1 37161450 10.1186/s13046-023-02686-1 PMC10170830

[95] Cheng R, Kong F, Tong L, Liu X, Xu K, Tang B (2018) Simultaneous detection of mitochondrial hydrogen selenide and superoxide anion in HepG2 cells under hypoxic conditions. Anal Chem 90:8116–8122. 10.1021/acs.analchem.8b01345 29879841 10.1021/acs.analchem.8b01345

[96] De Bem AF, Fiuza B, Calcerrada P, Brito PM, Peluffo G, Dinis TCP, Trujillo M, Rocha JBT, Radi R, Almeida LM (2013) Protective effect of diphenyl diselenide against peroxynitrite-mediated endothelial cell death: A comparison with ebselen. Nitric Oxide 31:20–30. 10.1016/j.niox.2013.03.003 23518198 10.1016/j.niox.2013.03.003

[97] Gallo-Rodriguez C, Rodriguez JB (2024) Organoselenium compounds in medicinal chemistry. ChemMedChem. 10.1002/cmdc.202400063 38778500 10.1002/cmdc.202400063

[98] Reeves MA, Hoffmann PR (2009) The human selenoproteome: Recent insights into functions and regulation. Cell Mol Life Sci 66:2457–2478. 10.1007/s00018-009-0032-4 19399585 10.1007/s00018-009-0032-4 PMC2866081

[99] Zhang Y, Roh YJ, Han SJ, Park I, Lee HM, Ok YS, Lee BC, Lee SR (2020) Role of selenoproteins in redox regulation of signaling and the antioxidant system: a review. Antioxidants 9:1–17. 10.3390/antiox9050383 10.3390/antiox9050383 PMC7278666 32380763

[100] Cardoso BR, Roberts BR, Bush AI, Hare DJ (2015) Selenium, selenoproteins and neurodegenerative diseases. Metallomics 7:1213–1228. ttps://doi.org/10.1039/c5mt00075k 25996565 10.1039/c5mt00075k

[101] Hatfield DL, Yoo MH, Carlson BA, Gladyshev VN (2009) Selenoproteins that function in cancer prevention and promotion. Biochim Biophys Acta Gen Subj 1790:1541–1545. 10.1016/j.bbagen.2009.03.001 10.1016/j.bbagen.2009.03.001 PMC2763959 19272412

[102] Díaz-Villanueva JF, Díaz-Molina R, García-González V (2015) Protein folding and mechanisms of proteostasis. Int J Mol Sci 16:17193–17230. 10.3390/ijms160817193 26225966 10.3390/ijms160817193 PMC4581189

[103] Liu Y, Xu C, Gu R, Han R, Li Z, Xu X (2024) Endoplasmic reticulum stress in diseases. MedComm 5:1–31. 10.1002/mco2.701 10.1002/mco2.701 PMC11345536 39188936

[104] Markouli M, Strepkos D, Papavassiliou AG, Piperi C (2020) Targeting of endoplasmic reticulum (ER) stress in gliomas. Pharmacol Res 157:104823. 10.1016/j.phrs.2020.104823 32305494 10.1016/j.phrs.2020.104823

[105] Schwarz DS, Blower MD (2016) The endoplasmic reticulum : structure, function and response to cellular signaling. Cell Mol Life Sci 73:79–94. 10.1007/s00018-015-2052-6 26433683 10.1007/s00018-015-2052-6 PMC4700099

[106] García-López D, Zaragoza-Ojeda M, Eguía-Aguilar P, Arenas-Huertero F (2024) Endoplasmic reticulum stress in gliomas: exploiting a dual-effect dysfunction through chemical pharmaceutical compounds and natural derivatives for therapeutical uses. Int J Mol Sci. 10.3390/ijms25074078 38612890 10.3390/ijms25074078 PMC11012637

[107] Hetz C, Axten JM, Patterson JB (2019) Pharmacological targeting of the unfolded protein response for disease intervention. Nat Chem Biol 15:764–775. 10.1038/s41589-019-0326-2 31320759 10.1038/s41589-019-0326-2

[108] Mu W, Zhi Y, Zhou J, Wang C, Chai K, Fan Z, Lv G (2024) Endoplasmic reticulum stress and quality control in relation to cisplatin resistance in tumor cells. Front Pharmacol 15:1–14. 10.3389/fphar.2024.1419468 10.3389/fphar.2024.1419468 PMC11211601 38948460

[109] Oakes SA (2020) Endoplasmic reticulum stress signaling in cancer cells. Am J Pathol 190:934–946. 10.1016/j.ajpath.2020.01.010 32112719 10.1016/j.ajpath.2020.01.010 PMC7237829

[110] Graner MW (2015) The unfolded protein response in glioblastomas: targetable or trouble? Future Sci OA. 10.4155/fso.15.45 28031873 10.4155/fso.15.45 PMC5137885

[111] Jehan C, Cartier D, Bucharles C, Anouar Y, Lihrmann I (2022) Emerging roles of ER-resident selenoproteins in brain physiology and physiopathology. Redox Biol. 10.1016/j.redox.2022.102412 35917681 10.1016/j.redox.2022.102412 PMC9344019

[112] Jia SZ, Xu XW, Zhang ZH, Chen C, Chen YB, Huang SL, Liu Q, Hoffmann PR, Song GL (2021) Selenoprotein K deficiency-induced apoptosis: a role for calpain and the ERS pathway. Redox Biol 47:102154. 10.1016/j.redox.2021.102154 34601426 10.1016/j.redox.2021.102154 PMC8495175

[113] Lee JH, Park KJ, Jang JK, Jeon YH, Ko KY, Kwon JH, Lee SR, Kim IY (2015) Selenoprotein S-dependent selenoprotein K binding to p97(VCP) protein is essential for endoplasmic reticulum-associated degradation. J Biol Chem 290:29941–29952. 10.1074/jbc.M115.680215 26504085 10.1074/jbc.M115.680215 PMC4706009

[114] Varlamova Patronymic-Gennadyevna E (2018) Participation of selenoproteins localized in the ER in the processes occurring in this organelle and in the regulation of carcinogenesis-associated processes. J Trace Elem Med Biol 48:172–180. 10.1016/j.jtemb.2018.04.005 29773177 10.1016/j.jtemb.2018.04.005

[115] Marfatiya S, Mubariz F, Pal A, RoyChoudhuri D, Mukherjee S, Maharajan N, Zalzman M, Banerjee A (2025) Targeting the unfolded protein response in cancer: exploiting endoplasmic reticulum stress for therapeutic intervention. Biochem Pharmacol 242:117221. 10.1016/j.bcp.2025.11722140789371 10.1016/j.bcp.2025.117221

[116] Unal B, Saatcioglu F (2025) Targeting the unfolded protein response for cancer therapy: mitigating tumor adaptation and immune suppression. Biomark Res 13:1–27. ttps://doi.org/10.1186/s40364-025-00813-y 41372991 10.1186/s40364-025-00813-y PMC12696942

[117] Walczak-Szeffer A, Piastowska-Ciesielska AW (2024) Endoplasmic reticulum stress as a target for retinoids in cancer treatment. Life Sci 352:122892. 10.1016/j.lfs.2024.122892 38971363 10.1016/j.lfs.2024.122892

[118] Bao L, Luo Q, Zhang J, Lao Z (2018) GRP78 overexpression as an unfavorable outcome in glioma patients. Int J Clin Exp Pathol 11:420–426 31938127 PMC6957966

[119] Suyama K, Watanabe M, Sakabe K, Okada Y, Matsuyama D, Kuroiwa M, Mochida J (2011) Overexpression of GRP78 protects glial cells from endoplasmic reticulum stress. Neurosci Lett 504:271–276. ttps://doi.org/10.1016/j.neulet.2011.09.045 21970967 10.1016/j.neulet.2011.09.045

[120] Tuncer C, Hacioglu C (2024) Borax induces ferroptosis of glioblastoma by targeting HSPA5/NRF2/GPx4/GSH pathways. J Cell Mol Med 28:1–13. 10.1111/jcmm.18206 10.1111/jcmm.18206 PMC10945083 38494858

[121] Zhang W, Shi Y, Oyang L, Cui S, Li S, Li J, Liu L, Li Y, Peng M, Tan S, Xia L, Lin J, Xu X, Wu N, Peng Q, Tang Y, Luo X, Liao Q, Jiang X, Zhou Y (2024) Endoplasmic reticulum stress—a key guardian in cancer. Cell Death Discov 10:1–16. 10.1038/s41420-024-02110-3 39080273 10.1038/s41420-024-02110-3 PMC11289465

[122] Kaufhold S, Bonavida B (2014) Central role of Snail1 in the regulation of EMT and resistance in cancer: a target for therapeutic intervention. J Exp Clin Cancer Res 33:1–19. 10.1186/s13046-014-0062-0 25084828 10.1186/s13046-014-0062-0 PMC4237825

[123] Kielbik M, Przygodzka P, Szulc-Kielbik I, Klink M (2023) Snail transcription factors as key regulators of chemoresistance, stemness and metastasis of ovarian cancer cells. Biochimica et Biophysica Acta (BBA) - Reviews on Cancer 1878:189003. 10.1016/j.bbcan.2023.189003 37863122 10.1016/j.bbcan.2023.189003

[124] Blahovcova E, Richterova R, Kolarovszki B, Dobrota D, Racay P, Hatok J (2015) Apoptosis-related gene expression in tumor tissue samples obtained from patients diagnosed with glioblastoma multiforme. Int J Mol Med 36:1677–1684. 10.3892/ijmm.2015.2369 26459752 10.3892/ijmm.2015.2369

[125] Lo Dico A, Martelli C, Diceglie C, Lucignani G, Ottobrini L (2018) Hypoxia-inducible factor-1α activity as a switch for glioblastoma responsiveness to temozolomide. Front Oncol. 10.3389/fonc.2018.00249 30013951 10.3389/fonc.2018.00249 PMC6036118

[126] Zhang L, Yang Y, Li Y, Wang C, Bian C, Wang H, Wang F (2025) Epigenetic regulation of histone modifications in glioblastoma: recent advances and therapeutic insights. Biomark Res 13:1–32. ttps://doi.org/10.1186/s40364-025-00788-w 40450300 10.1186/s40364-025-00788-w PMC12125905

[127] Baker BM, Boehling JR, Knopf S, Held S, Matossian M, Belgodere JA, Hoang VT, Collins-Burow BM, Martin EC, Lee SB, Burow ME, Drewry DH, Newman RH (2025) NEK family kinases: structure, function, and role in disease. Biomolecules 15:1–42. 10.3390/biom15101406 10.3390/biom15101406 PMC12564199 41154634

[128] Fry AM, O’Regan L, Sabir SR, Bayliss R (2012) Cell cycle regulation by the NEK family of protein kinases. J Cell Sci 125:4423–4433. 10.1242/jcs.111195 23132929 10.1242/jcs.111195 PMC3500863

[129] Nguyen K, Boehling J, Tran MN, Cheng T, Rivera A, Collins-Burow BM, Lee SB, Drewry DH, Burow ME (2023) NEK family review and correlations with patient survival outcomes in various cancer types. Cancers (Basel) 15:1–18. 10.3390/cancers15072067 10.3390/cancers15072067 PMC10093199 37046733

[130] Xiang J, Alafate W, Wu W, Wang Y, Li X, Xie W, Bai X, Li R, Wang M, Wang J (2022) NEK2 enhances malignancies of glioblastoma via NIK/NF-κB pathway. Cell Death Dis 13:1–13. 10.1038/s41419-022-04512-6 10.1038/s41419-022-04512-6 PMC8760305 35031599

[131] Zhu J, Cai Y, Liu P, Zhao W (2016) Frequent Nek1 overexpression in human gliomas. Biochem Biophys Res Commun 476:522–527. 10.1016/j.bbrc.2016.05.156 27251576 10.1016/j.bbrc.2016.05.156

[132] Xia J, Zhao H, Edmondson JL, Koss B, Zhan F (2025) Role of NEK2 in tumorigenesis and tumor progression. Trends Mol Med 31:79–93. 10.1016/j.molmed.2024.07.013 39181803 10.1016/j.molmed.2024.07.013 PMC11717647

[133] Tang X, Sui X, Weng L, Liu Y (2021) SNAIL1: Linking Tumor Metastasis to Immune Evasion. Front Immunol 12:1–11. 10.3389/fimmu.2021.724200 10.3389/fimmu.2021.724200 PMC8669501 34917071

[134] Kar F, Hacioğlu C, Kaçar S (2023) The dual role of boron *in vitro* neurotoxication of glioblastoma cells via SEMA3F/NRP2 and ferroptosis signaling pathways. Environ Toxicol 38:70–77. 10.1002/tox.23662 36136913 10.1002/tox.23662

[135] Mai A, Ye SW, Tu JY, Gao J, Kang ZF, Yao QM, Ting WJ (2023) Thymoquinone induces apoptosis in temozolomide-resistant glioblastoma cells via the p38 mitogen-activated protein kinase signaling pathway. Environ Toxicol 38:90–100. 10.1002/tox.23664 36176197 10.1002/tox.23664 PMC10087852

[136] Oliveira KA, Dal-Cim TA, Lopes FG, Nedel CB, Tasca CI (2017) Guanosine promotes cytotoxicity via adenosine receptors and induces apoptosis in temozolomide-treated A172 glioma cells. Purinergic Signal 13:305–318. 10.1007/s11302-017-9562-7 28536931 10.1007/s11302-017-9562-7 PMC5563291

[137] He L, Zhang L, Peng Y, He Z (2024) Selenium in cancer management: exploring the therapeutic potential. Front Oncol 14:1–24. 10.3389/fonc.2024.1490740 10.3389/fonc.2024.1490740 PMC11746096 39839762

[138] Letavayová L, Vlčková V, Brozmanová J (2006) Selenium: from cancer prevention to DNA damage. Toxicology 227:1–14. 10.1016/j.tox.2006.07.017 16935405 10.1016/j.tox.2006.07.017

[139] Wang K, Fu X, Li Y, Hou Y, Yang M, Sun J, Yi S, Fan C, Fu X, Zhai J, Sun B (2016) Induction of S-phase arrest in human glioma cells by selenocysteine, a natural selenium-containing agent via triggering reactive oxygen species-mediated DNA damage and modulating MAPKs and AKT pathways. Neurochem Res 41:1439–1447. 10.1007/s11064-016-1854-8 26846141 10.1007/s11064-016-1854-8

[140] Ibrahim M, Hur B, Mussulini M, Moro L, Assis AD, Rosemberg DB, Oliveira DD, Rocha JBT, Schwab RS, Henrique P, Souza DO, Rico EP (2014) Anxiolytic effects of diphenyl diselenide on adult zebra fi sh in a novelty paradigm. Prog Neuropsychopharmacol Biol Psychiatry 54:187–194. 10.1016/j.pnpbp.2014.06.002 24936773 10.1016/j.pnpbp.2014.06.002

[141] Liu Y, Beyer A, Aebersold R (2016) On the dependency of cellular protein levels on mRNA abundance. Cell 165:535–550. 10.1016/j.cell.2016.03.014 27104977 10.1016/j.cell.2016.03.014

[142] Yu R, Hao M, Liu A, Yang C, Xu P, Zhou Y, Tinkov AA, Yang A, Lv Q, Han Z, Wang C, Wang Z, Jiang J, Che X, Sun L, Zhou JC (2026) mRNA Rather than Protein Expression of Hepatic Selenoprotein H is More Sensitive to Short-term Low Aflatoxin B1 Exposure in Mice with Varying Selenium Intake. Biol Trace Elem Res. ttps://doi.org/10.1007/s12011-026-05003-x 10.1007/s12011-026-05003-x 41612114

